# Polymerisation‐Induced Self‐Assembly on Planar Surfaces: A New Approach for Controlling Surface Topography and Modulating Material‐Bio Interactions

**DOI:** 10.1002/anie.202507194

**Published:** 2025-08-14

**Authors:** Xin Xu, Jia‐Qi Xu, You‐Liang Zhu, Yixin Chang, Yuhao Zhang, Hui Peng, Zhong‐Yuan Lu, Andrew Whittaker, Changkui Fu

**Affiliations:** ^1^ Australian Institute for Bioengineering and Nanotechnology The University of Queensland St Lucia Queensland 4072 Australia; ^2^ State Key Laboratory of Supramolecular Structure and Materials College of Chemistry Jilin University Changchun 130012 China; ^3^ Australian Research Council Centre of Excellence for Green Electrochemical Transformation of Carbon Dioxide The University of Queensland St Lucia Queensland 4072 Australia

**Keywords:** Antibacterial, Antifouling, Polymer chemistry, Self‐assembly, Surface engineering

## Abstract

Polymerisation‐induced self‐assembly (PISA) has emerged as a highly efficient method for synthesising polymeric nanoparticles with diverse and well‐defined morphologies for a range of applications. While extensive research has focused on solution‐based PISA mediated by conformationally free macro‐stabilisers, the process of PISA on planar surfaces using surface‐tethered macro‐stabilisers with constrained mobility, namely surface PISA, remains largely unexplored. Investigating this process is significant to further advance PISA technology and expand its applications. In this work, we explore surface PISA through both experimental and computational approaches, revealing key differences from conventional solution‐based PISA. We also demonstrate that surface PISA offers an innovative approach for controlling surface topography and modulating material‐bio interactions. Specifically, we showcase its versatile application in creating slippery liquid‐infused porous surfaces (SLIPS) and encapsulating antibiotics, endowing material surfaces with enhanced antifouling and antimicrobial properties. We believe this work is a significant step forward for PISA technology and will create new opportunities for its broader applications.

## Graphical abstract

This work investigates surface‐initiated photopolymerisation‐induced self‐assembly (surface PISA) as an innovative strategy for directing nanostructure formation on planar surfaces, providing a new approach to tailoring material surface topography and achieving enhanced antifouling and antibacterial surface properties.

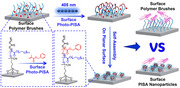

## Introduction

Polymerisation‐induced self‐assembly (PISA) has emerged as a highly efficient method to prepare polymeric nanoparticles with varied morphologies.^[^ ^1^, ^2^, ^3^, ^4^, ^5^, ^6^ ^]^ Compared to conventional post‐polymerisation block copolymer self‐assembly methods performed under dilute conditions (with solids content typically less than 1 wt%), PISA enables the preparation of polymeric nanoparticles at high solids content (10–50 wt%) while allowing for simple and precise control over the morphology of the nanoparticles.^[^ ^7^ ^]^ In a typical PISA process, a “living” solvophilic macro‐stabiliser is chain extended via the controlled polymerisation of core‐forming monomers. As the chain extension proceeds, block copolymers can form, grow and undergo spontaneous self‐assembly into nanoparticles in situ as the polymerisation proceeds. Depending on the degree of polymerisation (DP) of the formed solvophobic block of the core‐forming monomers, polymeric nanoparticles with diverse morphologies, such as spheres, worms, or vesicles, can be readily obtained. Over the past decade or so, numerous studies have been devoted to advancing PISA, highlighting a variety of parameters that influence the PISA process and the resulting nanoparticle morphologies. These include the solvent,^[^ ^8^, ^9^ ^]^ the chemical and structural characteristics of macro‐stabilisers,^[^ ^10^, ^11^ ^]^ the type and concentration of core‐forming monomers,^[^ ^12^, ^13^ ^]^ and even the polymerisation technologies used.^[^ ^14^ ^]^ Due to the ease and versatility for scale‐up synthesis of polymeric nanoparticles and precise control over nanoparticle morphology, PISA has been increasingly used across various fields, including drug delivery,^[^ ^15^, ^16^, ^17^, ^18^ ^]^ protein conjugates,^[^ ^19^, ^20^, ^21^, ^22^ ^]^ biomedical imaging,^[^ ^23^, ^24^, ^25^, ^26^ ^]^ artificial biology,^[^ ^27^, ^28^, ^29^ ^]^ hydrogels^[^ ^30^, ^31^ ^]^ and batteries.^[^ ^32^, ^33^ ^]^

**Scheme 1:**
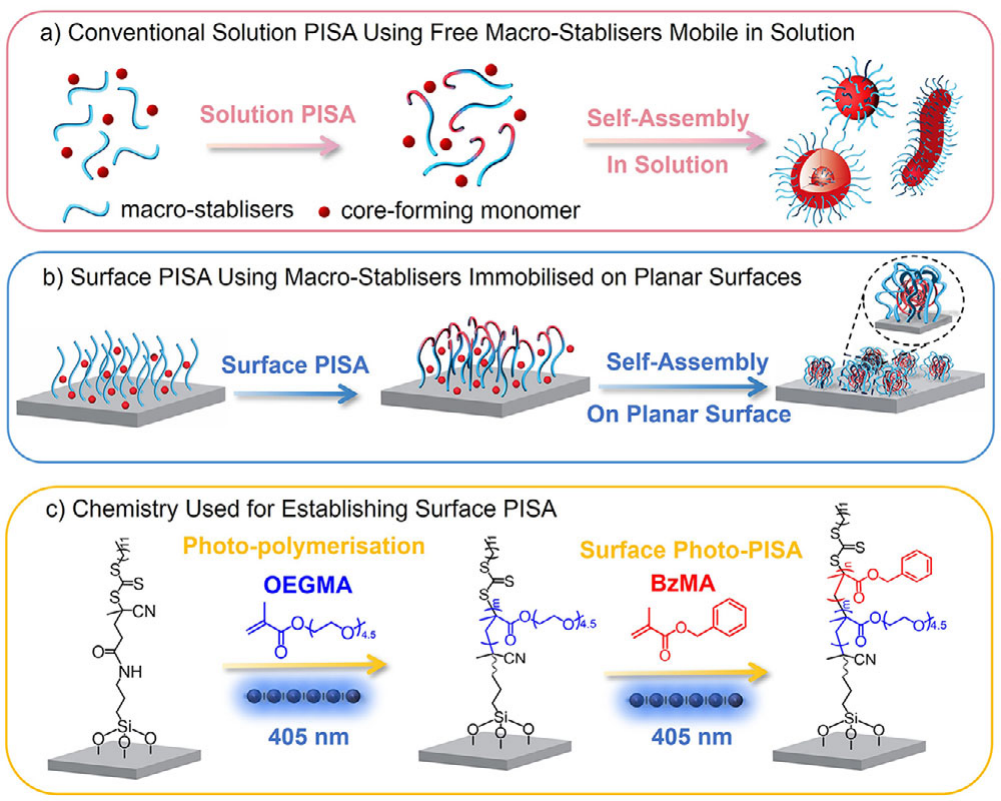
Schematic illustration of a) conventional solution PISA using free macro‐stabilisers, b) surface PISA mediated by surface‐tethered macro‐stabilisers immobilised on planar surfaces and c) the chemistry used in this work for establishing surface PISA.

Despite significant advances in the field of PISA, it is noted that the current PISA process is usually mediated by free macro‐stabilisers that are fully soluble in the solvent (denoted as solution PISA, Scheme 1a). The high mobility in solution of the free macro‐stabilisers effectively facilitates the self‐assembly and reorganisation of the growing block copolymers, enabling the formation of a variety of nanoparticle morphologies. On the other hand, the use of surface‐tethered macro‐stabilisers, immobilised on material surfaces with constrained chain mobility, has rarely been explored in PISA (referred to as surface PISA, Scheme 1b). It is expected that the constrained mobility of the surface‐tethered macro‐stabilisers could profoundly impact the PISA process, potentially leading to different kinetics of nanoparticle formation and morphology transition behaviour compared to those observed in conventional solution PISA. Several prior studies have investigated surface PISA using macro‐stabilisers immobilised on the surface of nanoparticles.^[^ ^34^, ^35^, ^36^, ^37^ ^]^ Nanostructures could be successfully formed on the surface of nanoparticles, leading to organic–inorganic hybrid nanomaterials. Recently, Niu et al. reported another example of surface PISA.^[^ ^38^ ^]^ In this seminal work, they used macro‐stabilisers tethered on a planar surface to mediate PISA with the addition of free macro‐stabilisers. Typical nanoparticle morphologies such as spheres, worms and vesicles were formed on the planar surface, akin to those seen in the solution PISA using free macro‐stabilisers. Nonetheless, the added free macro‐stabilisers, present in a significantly greater amount than the surface‐tethered macro‐stabilisers, dominate the PISA process. Thus, it remains poorly understood how a PISA process, exclusively mediated by surface‐tethered macro‐stabilisers, occurs and proceeds on a planar surface.

Surface topography plays an important role in determining how material surfaces interact with biological systems.^[^ ^39^, ^40^, ^41^, ^42^ ^]^ Micro‐ or nanostructured surfaces exhibit increased surface roughness compared to flat surfaces without topography features, demonstrating an enhanced ability to modulate biological responses.^[^ ^43^, ^44^, ^45^, ^46^, ^47^, ^48^, ^49^, ^50^ ^]^ Control over surface topography therefore allows for the tailored design of surface properties to effectively modulate material‐bio interactions, particularly in repelling unwanted biological adhesion, i.e., antifouling properties. Surface polymer brushes represent an effective strategy for achieving antifouling properties.^[^ ^51^, ^52^, ^53^, ^54^ ^]^ These brushes are created by tethering polymer chains to a substrate,^[^ ^55^, ^56^, ^57^, ^58^ ^]^ forming a dense layer that effectively prevents biological adhesion. However, current surface polymer brushes typically lack topographical features. While the inherent chemical/physical properties, such as hydrophilicity or low surface energy, of the surface polymer brushes contribute to antifouling properties, incorporating topographical features could further enhance their effectiveness in antifouling applications.

In this work, we report surface PISA exclusively mediated by surface‐tethered macro‐stabilisers immobilised on planar surfaces (Scheme 1b). Taking advantage of the ease of surface‐initiated photoinduced electron transfer‐reversible addition‐fragmentation chain transfer (PET‐RAFT) polymerisation,^[^ ^59^ ^]^ we investigated the effect of several key parameters, including the chain extension time, the grafting density and the chain length of the surface‐tethered macro‐stabilisers, as well as the concentration of the core‐forming monomer, on the formation of polymeric nanoparticles on planar surfaces. The study highlights the remarked difference between surface PISA and conventional solution PISA. Furthermore, we demonstrated that surface PISA can serve as a new approach for controlling material surface topography and modulating material‐bio interactions to achieve superior antifouling properties compared to conventional surface polymer brushes without topographical features. We also explored the use of surface PISA to develop slippery liquid‐infused porous surfaces (SLIPS),^[^ ^60^ ^]^ a cutting‐edge antifouling technology. Finally, we show that antibiotics can be easily encapsulated within the nanoparticles formed on planar surfaces via the surface PISA approach, endowing the surfaces with bactericidal properties. We believe this work significantly broadens the scope of PISA, transitioning its application from traditional solution scenarios to surface‐based functionalisation, enabling the creation of advanced materials with precisely tailored surface properties for a wide range of applications.

## Results and Discussion

### Synthesis of Surface‐Tethered POEGMA Macro‐Stabiliser

In this work, we chose oligo(ethylene glycol) methyl ether methacrylate (OEGMA, *M*_n_ = 300 g mol^−1^) and benzyl methacrylate (BzMA) as the macro‐stabiliser monomer and the core‐forming monomer, respectively, for the investigation of the surface PISA process in alcoholic conditions (Scheme 1c). This PISA system has been extensively investigated and is known to be able to form polymeric nanoparticles with different morphologies in solution, as demonstrated in prior research^[^ ^61^, ^62^, ^63^, ^64^ ^]^ and in a solution PISA we conducted (Figure S1). Silicon wafers were selected as the planar surface substrates and were first treated with sodium hydroxide solution to produce a surface layer of hydroxyl groups (Scheme S1). Afterwards, the substrates were decorated with amine groups by surface condensation of 3‐aminopropyltriethoxysilane (APTES). The successful introduction of amine groups on the surface of the silicon wafers was confirmed by X‐ray photoelectron spectroscopy (XPS) analysis, showing the presence of a nitrogen peak (N 1s) at 400 eV (Figure S2). Then, a RAFT agent, 4‐cyano‐4‐[(dodecylsulfanylthiocarbonyl)sulfanyl]pentanoic acid (CDTPA), was attached to the amine‐modified silicon wafers. The successful attachment of CDTPA was again verified by XPS, showing the characteristic peaks at 229.3 eV (S 2s) and at 165.3 eV (S 2p) due to the trithiocarbonate groups (Figure S2) and the peaks at 285.6 eV (C 1s) and 396.2 eV (N 1s) due to the cyano groups of CDTPA (Figure S3). The CDTPA‐modified surfaces were used to grow POEGMA brushes as the surface‐tethered macro‐stabilisers for PISA (Scheme 1c). Surface‐initiated PET‐RAFT polymerisation of OEGMA was conducted in the presence of free CDTPA under the irradiation of LED light (λ_m_ = 405 nm; maximum irradiance = 3.5 mW cm^−2^ at 5 cm distance) with a ratio of [CDTPA]:[OEGMA] = 1:50 using Eosin Y as a photo‐redox catalyst.^[^ ^65^, ^66^ ^]^ After polymerisation for 18 h, the conversion of OEGMA reached 71% (Table S1). The generated free POEGMA was collected and characterised by ^1^H nuclear magnetic resonance (NMR) (Figure S4) and size‐exclusion chromatography (SEC) as an approximate model for the surface‐tethered POEGMA brushes. The soluble POEGMA had a DP of 35, a molecular weight (*M*_n_) of 11 930 g mol^−1^, and a molecular weight dispersity (*Đ*) of 1.38 (Table S1). The successful synthesis of surface‐tethered POEGMA was further confirmed by XPS (Figure S1), evidenced by an increase in the surface carbon content (Table S2) and the presence of new C─O bonds (Figure S5), compared to the CDTPA‐modified surface before polymerisation.

### Effect of Time‐Kinetics of Surface PISA

**Figure 1:**
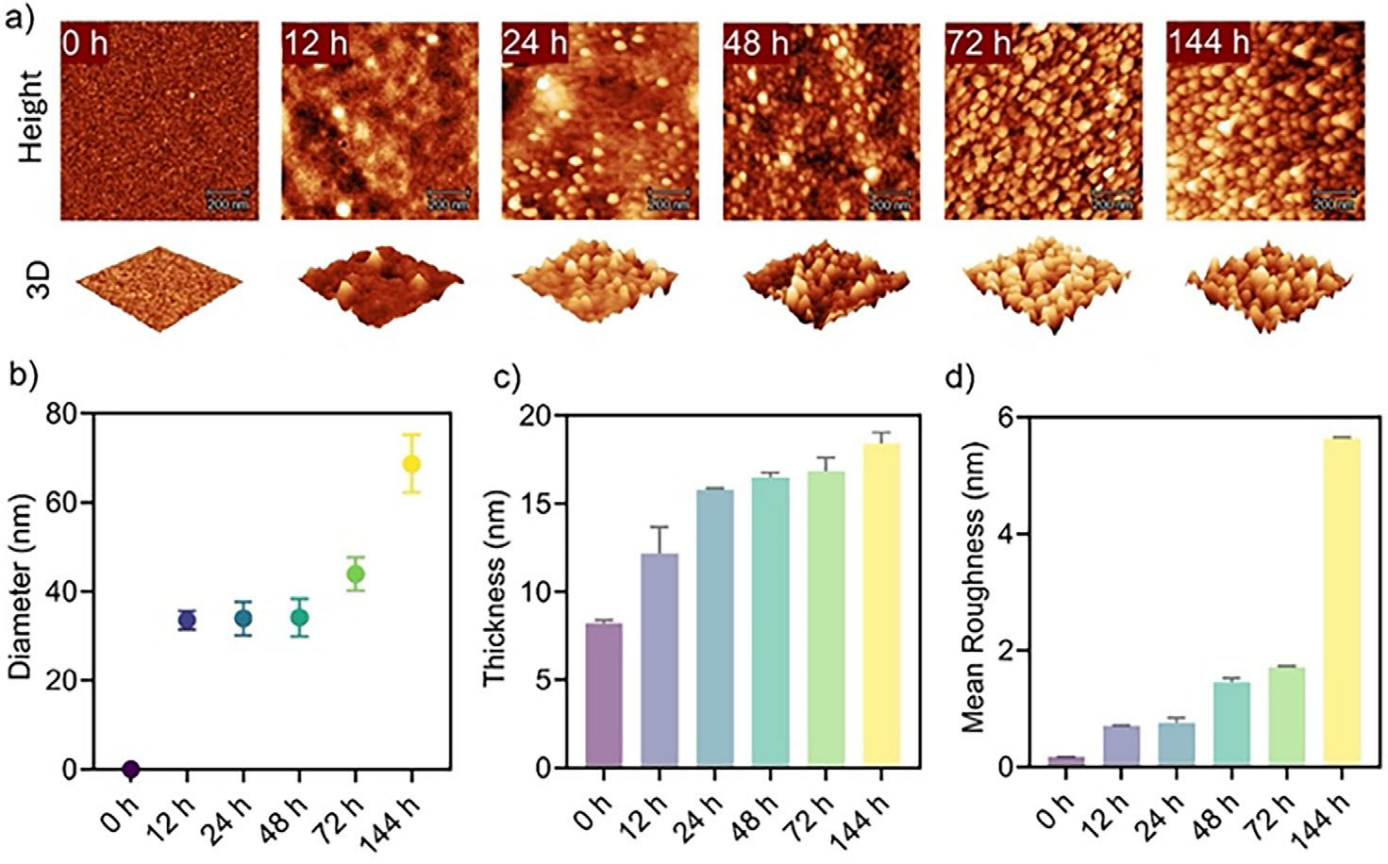
a) AFM height and 3D images of the silicon wafer surfaces after surface PISA for different times (scale bar: 200 nm), b) particle diameter, c) film thickness and d) mean surface roughness of the polymeric nanoparticles formed via surface PISA on the silicon wafers.

We next investigated the photo‐PISA on a planar surface using the surface‐tethered POEGMA brushes as macro‐stabilisers. The POEGMA‐grafted silicon wafers were immersed in an ethanol solution containing BzMA (175 mg mL^−1^) and a photo‐redox catalyst, Eosin Y (0.075 mg mL^−1^), which were irradiated under LED light at room temperature. Atomic force microscopy (AFM) and ellipsometery were used to monitor the changes occurring on the surfaces over time. Note that these measurements were of samples after drying, so some features solvated during the surface PISA process may appear flattened after drying. At 0 h before the polymerisation, the POEGMA‐grafted silicon wafer showed a smooth surface with minimal topographical features (Figure 1a,b). The POEGMA layer had a thickness of approximately 8.2 nm measured by ellipsometry (Figure 1c) and a mean surface roughness of 0.17 nm measured by AFM (Figure 1d). After polymerisation for 12 h, sparse nanoparticles with a diameter of ∼34.5 nm were found to form on the surface (Figure 1a), accompanied by an increase in the thickness of the polymer layer from 8.2 to 12.2 nm (Figure 1c) as well as a notable increase in the surface roughness from 0.17 to 0.70 nm (Figure 1d). As the polymerisation time further extended, the formation of nanoparticles became more pronounced, leading to an increased density of nanoparticles on the surface (Figure 1a). After 144 h polymerisation, the size, thickness and surface roughness of the formed nanoparticles increased gradually to 68.6 nm (Figure 1b), 18.4 nm (Figure 1c) and 5.63 nm (Figure 1d), respectively. XPS was used to confirm the successful chain extension of surface‐tethered POEGMA brushes by PET‐RAFT polymerisation of BzMA, showing the presence of an aromatic C═C peak due to the BzMA content (Figure S6). To further validate that the formation of nanoparticles arose from a self‐assembly process occurring on the planar surface, the surface nanoparticles obtained after 48 h polymerisation were treated by tetrahydrofuran (THF), a good solvent for both POEGMA and PBzMA blocks. As shown in Figure S7a, after THF treatment, no nanoparticles were observed on the surface of the silicon wafer, owing to the disassembly of the nanoparticles into surface polymer brushes. However, once the surface polymer brushes were re‐treated with ethanol, the nanoparticles were regenerated on the surface with similar sizes (Figure S7b), indicating that the original surface nanoparticles were indeed produced via a surface PISA process. It is also notable that the surface nanoparticles formed by ethanol retreatment showed a greater thickness and significantly increased surface roughness compared to the original surface nanoparticles formed by surface PISA (Figure S7c,d). A further five‐cycle alternating treatment with THF and ethanol confirmed the observed increase in surface roughness (Figure S7e). The lower surface roughness observed in surface PISA suggests a more uniform and compact self‐assembly process, resulting from the in situ formation of nanoparticles directly on the surface during polymerisation, compared to the post‐polymerisation assembly of surface‐grafted block copolymers.

In addition, we evaluated the stability of the surface PISA nanoparticles by alternately immersing the wafers in ethanol and water, each for 1 day, over a 7‐day period to assess their resistance to solvent‐induced detachment. This treatment was designed to mimic repeated solvent exposure and test the robustness of nanoparticle attachment under fluctuating solvent environments. Following this treatment, the nanoparticles were re‐examined by AFM. As shown in Figure S8, the surface nanoparticles retained their original morphology, size and surface density, indicating strong anchoring to the substrate and minimal particle loss. A slight increase in surface roughness was observed, from ∼1.21 to ∼1.64 nm compared to the untreated surfaces. This modest change may be possibly due to swelling or conformational adjustments of the nanoparticles in response to alternating solvent exposure. Overall, these results confirm the structural stability of surface PISA nanoparticles under solvent stress and highlight their potential for long‐term application in dynamic environments.

To further test the general applicability of surface PISA, diacetone acrylamide (DAAM),^[^ ^67^, ^68^ ^]^ another commonly‐used core‐forming monomer in solution PISA, was used as an alternative to BzMA. Surface‐initiated PET‐RAFT polymerisation was performed in aqueous solution using the surface‐tethered POEGMA and DAAM. Nanoparticles were also successfully formed on the planar surface (Figure S9). We further conducted surface PISA using poly(*N,N*‐dimethyl acrylamide) (PDMA) as the macro‐CTA and styrene (St) as the core‐forming monomer. Again, nanoparticles were successfully formed on the surface (Figure S10), demonstrating the universality of the surface PISA process. Interestingly, in all the surface PISA processes, only spherical nanoparticles were produced regardless of the reaction time, macro‐CTAs and core‐forming monomers, with no other morphologies such as worms observed. Although higher‐order nanoparticle morphologies are not always formed in conventional solution PISA, the absence of these morphologies in surface PISA is more likely attributed to the constrained chain mobility of the surface‐tethered block copolymers. This limitation in chain mobility restricts their ability to reorganise into different morphologies during the chain extension process, which contrasts with the observed typical morphological evolution in solution PISA. Nonetheless, some nanoparticles displayed elongated and merged shapes (Figure S11). This may suggest a tendency of nanoparticle fusion to develop higher‐order morphologies, which, however, is restricted by the mobility constraints of tethered polymer chains. Finally, the presence of nanoparticles significantly alters the surface topography of the silicon wafers, transforming a smooth, flat surface into a rough, complex one with highly defined 3D topographical features (Figure 1a). By controlling the reaction time of surface PISA, the surface roughness and topography of the planar surfaces can be tailored (Figure 1d), which has the potential to modulate the interaction of the material surface with biological systems and enable advanced biological applications (vide infra).

### Effect of the Concentration of the Core‐Forming Monomer on Surface PISA

**Figure 2:**
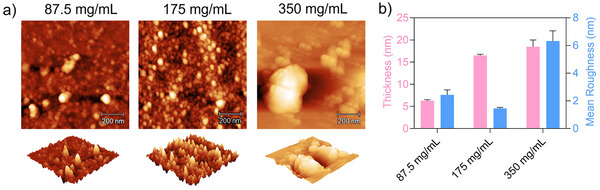
a) AFM images and b) film thickness and mean roughness of silicon wafers via surface PISA for 48 h using different concentrations of BzMA (87.5, 175, 350 mg mL^−1^).

The concentration of core‐forming monomer can potentially affect the surface PISA process and the characteristics of formed surface nanoparticles. To study this effect, three concentrations of BzMA (87.5, 175 and 350 mg mL^−1^) were used for conducting surface PISA. The AFM images of the wafers after surface PISA are shown in Figure 2a. For all the concentrations, spherical nanoparticles were formed on the surface, and the thickness of the surface nanoparticles increased when a higher concentration of BzMA was used (Figure 2b). The concentration of BzMA also affects the density of nanoparticles formed on the surfaces. For instance, a higher density of surface nanoparticles was formed using 175 mg mL^−1^ of BzMA compared to using 87.5 mg mL^−1^. The higher density of nanoparticles with more uniform size produces a smoother surface evidenced by a lower surface mean roughness (Figure 2b). On further increasing the concentration of BzMA to 350 mg mL^−1^, nanoparticles with large size and irregular morphologies were formed, along with nanoparticles of smaller sizes (Figure 2a). The nonuniformity in size and morphology might be attributed to the increased occurrence of polymer chain coupling and termination during chain extension at this concentration, resulting in a partial loss of control over the polymerisation during the surface PISA process. As a result, a rougher surface (surface roughness = 6.3 nm) was formed (Figure 2b).

### Effect of the Grafting Density of Surface‐Tethered POEGMA on Surface PISA

**Figure 3:**
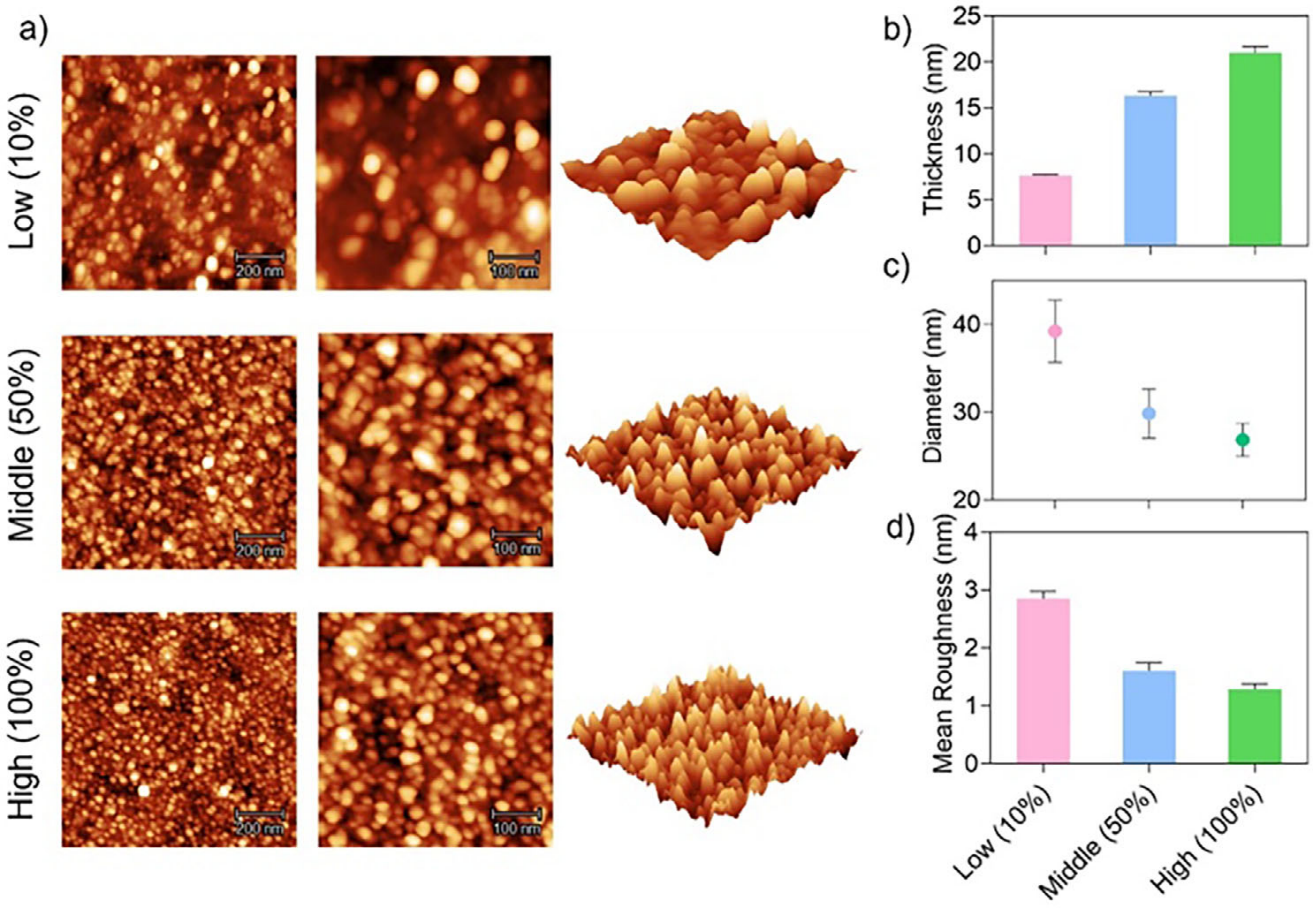
a) AFM images, b) film thickness, c) particle diameter and d) mean roughness of surface PISA using surface‐tethered POEGMA with low, middle and high grafting density.

The effect of the grafting density of surface‐tethered POEGMA on the formation of surface nanoparticles was also investigated. To achieve different grafting densities, we treated the silicon wafers with varying ratios (100%:0%, 50%:50%, 10%:90%) of APTES, which contains an NHS‐reactive amine group, and 3‐(triethoxysilyl)propionitrile (TESPN), which contains a non‐NHS‐reactive cyano group, to afford surfaces functionalised with different amounts of amine groups (Scheme S2). This varying degree of amine functionalisation was confirmed by XPS (Figure S12). The amine‐functionalised surfaces were further modified with CDTPA, then used to prepare surface‐tethered POEGMA brushes with different grafting densities via surface‐initiated PET‐RAFT polymerisation (Scheme S2). The grafting density of the surface POEGMA brushes prepared using the silicon wafers treated with 10%:90%, 50%:50% and 100%:0% ratios of APTES and TESPN was denoted as low (10%), middle (50%) and high (100%), respectively. These surface‐tethered POEGMA brushes were characterised by AFM and ellipsometry, revealing minimal surface topographical features (Figure S13a) and slightly increased thickness as the grafting density increased (Figure S13b). Their grafting densities were further measured by ellipsometry to be 0.14, 0.20 and 0.28 chains nm^−2^ (Figure S14). Using the surface‐tethered POEGMA brushes with different grafting densities, surface PISA was conducted with 175 mg mL^−1^ of BzMA over a polymerisation time of 48 h followed by AFM and ellipsometry characterisation. At all the grafting densities, nanoparticles could be successfully formed on the surfaces (Figure 3a). The thickness of the nanoparticles increased on increasing the grafting density (Figure 3b). On the other hand, the size of the formed nanoparticles decreased, changing from ∼39.2 nm at low grafting density to ∼29.9 and ∼26.9 nm at middle and high grafting densities (Figure 3c). Meanwhile, the nanoparticles were more densely packed and exhibited greater uniformity at a higher grafting density compared to a lower grafting density, leading to smoother nanoparticle coating with reduced surface roughness (Figure 3d). Further power spectral density analysis based on the AFM images of the surface PISA confirmed this correlation between the grafting density and surface smoothness (Figure S15).

### Effect of the Chain Length of Surface‐Tethered POEGMA on Surface PISA

**Figure 4:**
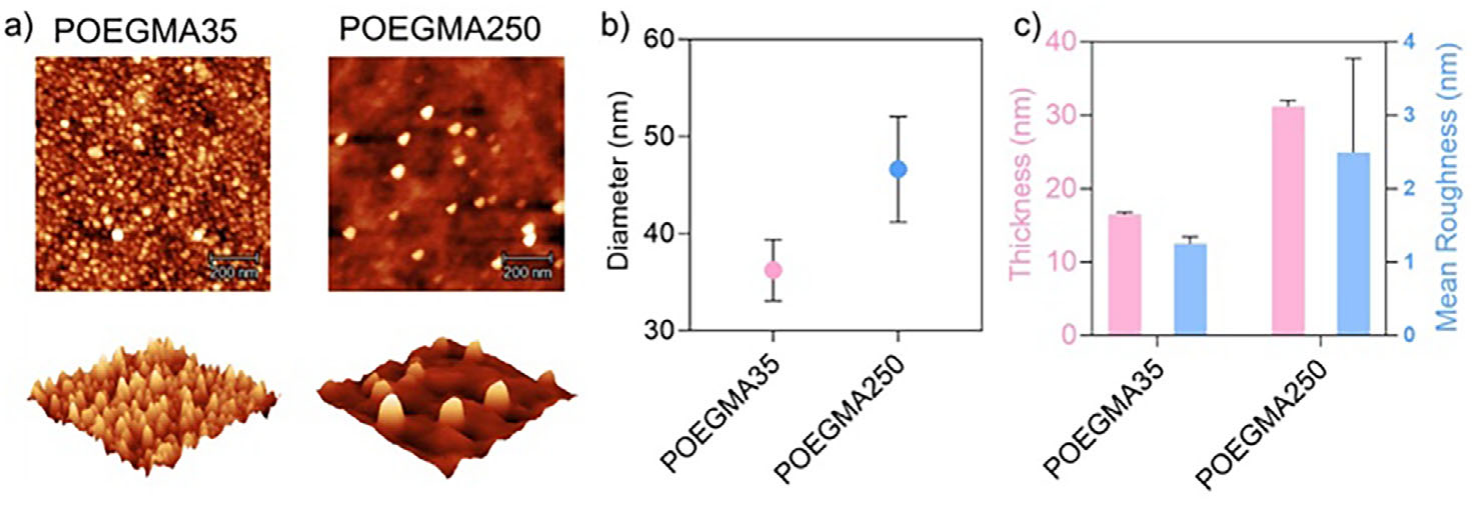
a) AFM images, b) particle diameter and c) film thickness and surface roughness of surface PISA mediated by surface‐tethered POEGMA with different chain lengths (DP35 and DP250) after 48 h polymerisation.

We further investigated the effect of the chain length of the surface‐tethered POEGMA on surface PISA. Surface‐tethered POEGMA with a DP of 35 or a DP of 250 were used for surface PISA with 175 mg mL^−1^ of BzMA. The AFM images of the wafers after 48 h surface PISA are shown in Figure 4a. It can be seen that the surface grafted with shorter POEGMA chains (DP35) led to the formation of smaller, more densely packed and more uniform nanoparticles with an average diameter of ∼36 nm (Figure 4b). In contrast, the surface grafted with longer POEGMA chains (DP250, *M*_n_ = 69 340 g mol^−1^, *Đ* = 1.56, Table S1) formed a low density of nanoparticles with a larger diameter of ∼46 nm (Figure 4a,b). Meanwhile, an increase in the thickness and surface mean roughness was observed (Figure 4c). The lower density of nanoparticles formed with the longer POEGMA is understandable. The formation of nanoparticles requires the packing ratio of the POEGMA and PBzMA blocks to exceed a certain point. The longer surface‐tethered POEGMA needs a longer block of PBzMA to self‐assemble into nanoparticles. A polymerisation time of 48 h appeared insufficient to form a PBzMA block of length adequate to drive the self‐assembly of all of the block copolymers. Given the relatively broad molecular weight dispersity (*Đ* = 1.56) of the longer surface‐tethered POEGMA250 (Table S1), it is therefore reasoned that the shorter surface‐tethered POEGMA brushes formed nanoparticles first within the 48‐h period of surface PISA, while those longer surface‐tethered POEGMA brushes, although chain extended, formed block copolymers that remained fully solvated in the solvent and did not undergo self‐assembly. This could explain the formation of a low density of nanoparticles when using the longer surface‐tethered POEGMA250 as macro‐stabilisers after surface PISA for 48 h. On further increasing the reaction time to 144 h, more nanoparticles were formed (Figure S16), suggesting the continued growth and self‐assembly of the block copolymers into nanoparticles once the second PBzMA block reached a sufficient chain length.

### Dissipative Particle Dynamics Simulation of Surface PISA

**Figure 5:**
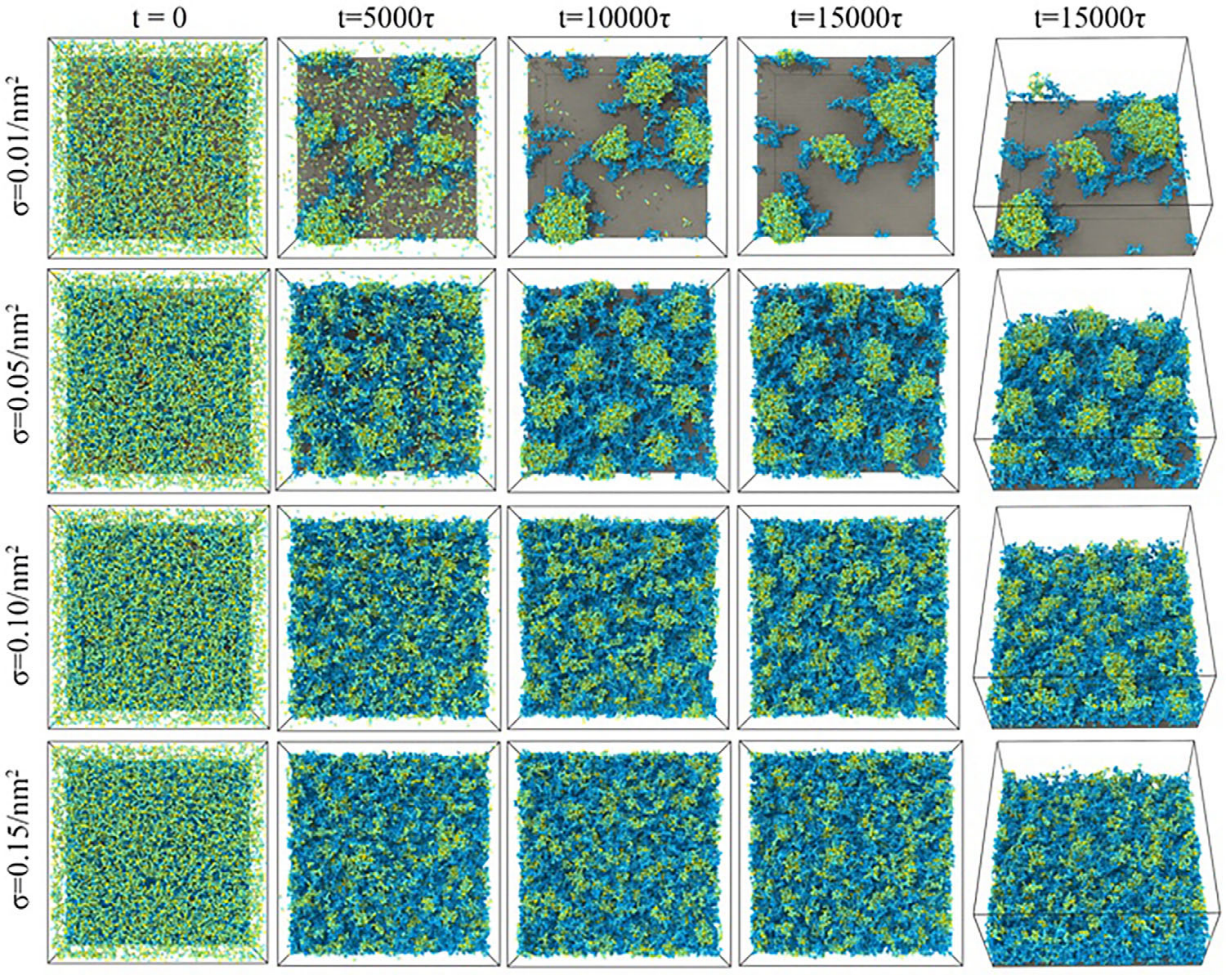
Time‐dependent snapshots of the DPD simulations of surface PISA at varying grafting densities of POEGMA. Cyan: POEGMA; yellow–green: BzMA or PBzMA. The solvent is not shown for clarity.

Dissipative particle dynamics (DPD) simulation has proven to be a powerful method to computationally study the self‐assembly of block copolymers.^[^ ^69^, ^70^ ^]^ To further understand how surface PISA proceeds, we used DPD simulations to investigate the chain extension from surface‐tethered macro‐stabilisers and the self‐assembly of the growing block copolymers on a planar surface. In our DPD simulations, we represent the monomer OEGMA with three connected beads and the core‐forming monomer BzMA with two connected beads, allowing for a more accurate depiction of their molecular size and topological features (Figure S17). Surface‐tethered POEGMA brushes (DP = 35) with varying grafting densities of 0.01, 0.05, 0.10 and 0.15 chains nm^−2^ were established, then the chain was extended via the polymerisation of BzMA, with the initial number of BzMA molecules set to 5000. The entire process of the chain extension and self‐assembly of the growing block copolymers was recorded (Figure S18 and Videos S1–S4). Snapshots of the DPD simulations at four representative time points are presented in Figure 5, in which the cyan represents POEGMA and the yellow–green represents BzMA or PBzMA. The DPD simulations clearly revealed the spontaneous self‐assembly process of the growing surface‐tethered POEGMA‐*b*‐PBzMA block copolymers, induced by the chain extension polymerisation of BzMA, resulting in the formation of nanoparticles on the planar surfaces. The size of the nanoparticles increased as the chain extension proceeded, in agreement with the experimental results (Figure 1b). Under the current simulation conditions, spherical nanoparticles were formed throughout the entire simulation process regardless of chain length of the second block of PBzMA (Figure S18) and the grafting density of POEGMA. However, merging of nanoparticles can be observed as chain extension progressed (Figure 5). The grafting density of the surface‐tethered POEGMA exhibited a significant impact on the size, density and surface roughness of the resulting nanoparticles. Higher grafting densities resulted in the formation of smaller nanoparticles (Figure S19) with increased density and reduced surface roughness, similar to the experimental observations (Figure 3c,d). Moreover, the grafting density of POEGMA greatly impacts the surface coverage of the nanoparticles. Insufficient coverage of the PBzMA core by POEGMA shells is observed at lower grafting densities compared to higher grafting densities (Figure 5), leading to greater exposure of the PBzMA core to the environment, which may impact the surface properties. This will be discussed in the next section.

### Antifouling Properties of Surfaces Modified by Surface PISA

**Figure 6:**
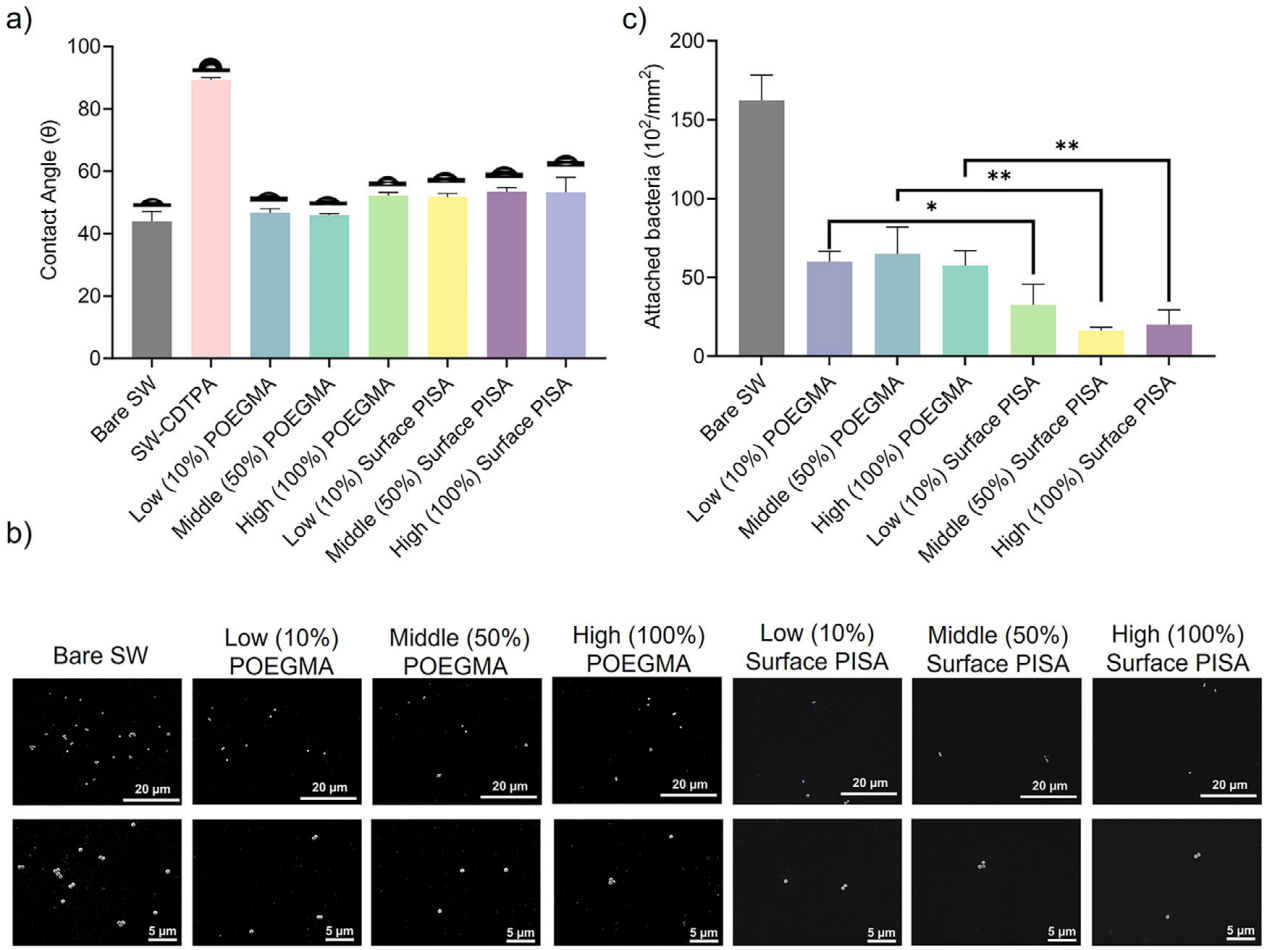
a) Water contact angle of silicon wafer surfaces modified by different approaches, b) SEM images (scale bar: top panel 20 µm, bottom panel: 5 µm) of *S. aureus* adhered to the silicon wafer surfaces and c) number of *S. aureus* adhered to these surfaces. **P ≤ 0.01, *P ≤ 0.05, *n* = 3.

The application of polymer brush coatings on material surfaces represents an effective antifouling strategy to prevent unwanted biological adhesion.^[^ ^52^ ^]^ Prior research has highlighted various chemical and structural parameters of surface polymer brushes, such as their chemical nature, grafting density, coating thickness and molecular topology, which can influence their antifouling performance.^[^ ^51^ ^]^ However, the effect of surface topography of polymer brushes on antifouling properties remains largely unexplored due to a lack of technologies that can precisely and readily control the surface topography of polymer coatings. In this work, we sought to use surface PISA to create defined surface topography of polymer brushes and investigated the effect of this topography on material surface properties and material‐bio interactions. The wettability of polymer brush coatings plays a crucial role in determining their surface properties and was therefore evaluated first. The water contact angle was measured for various surfaces, including Bare silicon wafer (Bare SW), silicon wafer modified with CDTPA (SW‐CDTPA), silicon wafers modified with POEGMA of different grafting densities (designated as low (10%), middle (50%) and high (100%) POEGMA), and silicon wafers modified by surface PISA (designated as low (10%), middle (50%) and high (100%) surface PISA) (Figure 6a). The Bare SW showed a contact angle of 44°, which increased to 89° after modification with hydrophobic CDTPA. The modification of hydrophilic POEGMA significantly decreased the surface contact angle to 46°–52°. The contact angle slightly increased when modified with higher grafting densities of POEGMA, correlating with the greater presence of hydrophobic CDTPA (Figure S20). The low‐ and middle‐grafting density surface PISA‐modified silicon wafers exhibited slightly larger contact angles compared to their corresponding POEGMA‐modified silicon wafers (Figure 6a), likely due to minor exposure of the hydrophobic PBzMA core resulting from insufficient POEGMA coverage at lower grafting densities, as indicated by the DPD simulations (Figure 5). However, at the high grafting density, the surface PISA‐modified silicon wafer exhibited a contact angle similar to that of the POEGMA‐modified silicon wafer. This indicated that the hydrophobic PBzMA core of the polymeric nanoparticles, formed at high grafting density, was completely covered by POEGMA, thereby effectively shielding it from environmental exposure that could influence surface wettability.

We then assessed the antifouling properties of the surfaces by investigating their capability to resist bacterial adhesion. The silicon wafers were incubated in a suspension of *Staphylococcus aureus* (*S. aureus*) for 10 h, then removed, washed and characterised by scanning electron microscopy (SEM). As shown in Figures 6b and S21, a large number of *S. aureus* cells, up to 1.62 × 10^4^ mm^−^^2^ (Figure 6c), was found on the surface of the unmodified Bare SWs, indicating its poor ability to resist bacterial adhesion. In contrast, the silicon wafers modified with POEGMA brushes possess a strong surface hydration layer, enhancing their ability to resist bacterial adhesion, with a significantly reduced number of *S. aureus* adhering to the surfaces. The surface PISA‐modified silicon wafers exhibited a lower number of adhered bacterial cells compared with those only grafted with POEGMA polymer brushes, across all grafting densities (Figure 6c). This improved ability to resist bacterial adhesion can be attributed to the nanoscale surface topography created by the densely packed nanoparticles by surface PISA. The complex 3D surface topography increased the surface roughness (1.28–2.85 nm, Figure 2d) compared to those modified with POEGMA brushes with minimal topographical features and low surface roughness (0.17 nm, Figure 1d). The increased surface roughness can reduce the entropic and enthalpic driving forces for bacterial adhesion, thereby enhancing the surface's antifouling properties, as demonstrated in several prior studies.^[^ ^71^, ^72^, ^73^ ^]^ In addition, since one end is anchored to the surface, the POEGMA block adopts a “loop” structure after surface PISA (Scheme 1b). This “loop” structure can generate strong steric repulsion, thus may also contribute to improved antifouling properties.^[^ ^74^, ^75^, ^76^, ^77^ ^]^ It is also notable that the silicon wafer modified with surface PISA at the middle grafting density exhibited the lowest level of bacterial adhesion compared to those modified at low or high grafting densities (Figure 6c).

To further validate the influence of surface topography on antifouling properties, we prepared two surfaces with distinct nanoparticle densities via surface PISA (Figure S22a) and subsequently assessed their contact angles and surface roughness (Figure S22b,c) and quantified bacterial adhesion (Figure S22d,e). The results show that nanoparticle density has minimal effect on the surface contact angles. This observation is understandable and consistent with the results shown in Figure 6a, as all surfaces were coated with POEGMA, which primarily determines surface wettability. However, we observed that the surface with a higher nanoparticle density (636 nanoparticles µm^−2^) and lower surface roughness exhibited significantly lower bacterial adhesion compared to the surface with a lower nanoparticle density (288 nanoparticles µm^−2^) and higher surface roughness (Figure S22d,e). This indicates that, despite similar wettability, increased surface topographical complexity driven by higher nanoparticle density can significantly reduce bacterial attachment by limiting effective contact area and introducing nanoscale physical barriers. This observation highlights the importance and potential of precisely controlling surface topography through surface PISA to modulate material‐bio interactions and achieve optimal antifouling properties.

### Fabrication of SLIPS Based on Surface PISA

**Figure 7:**
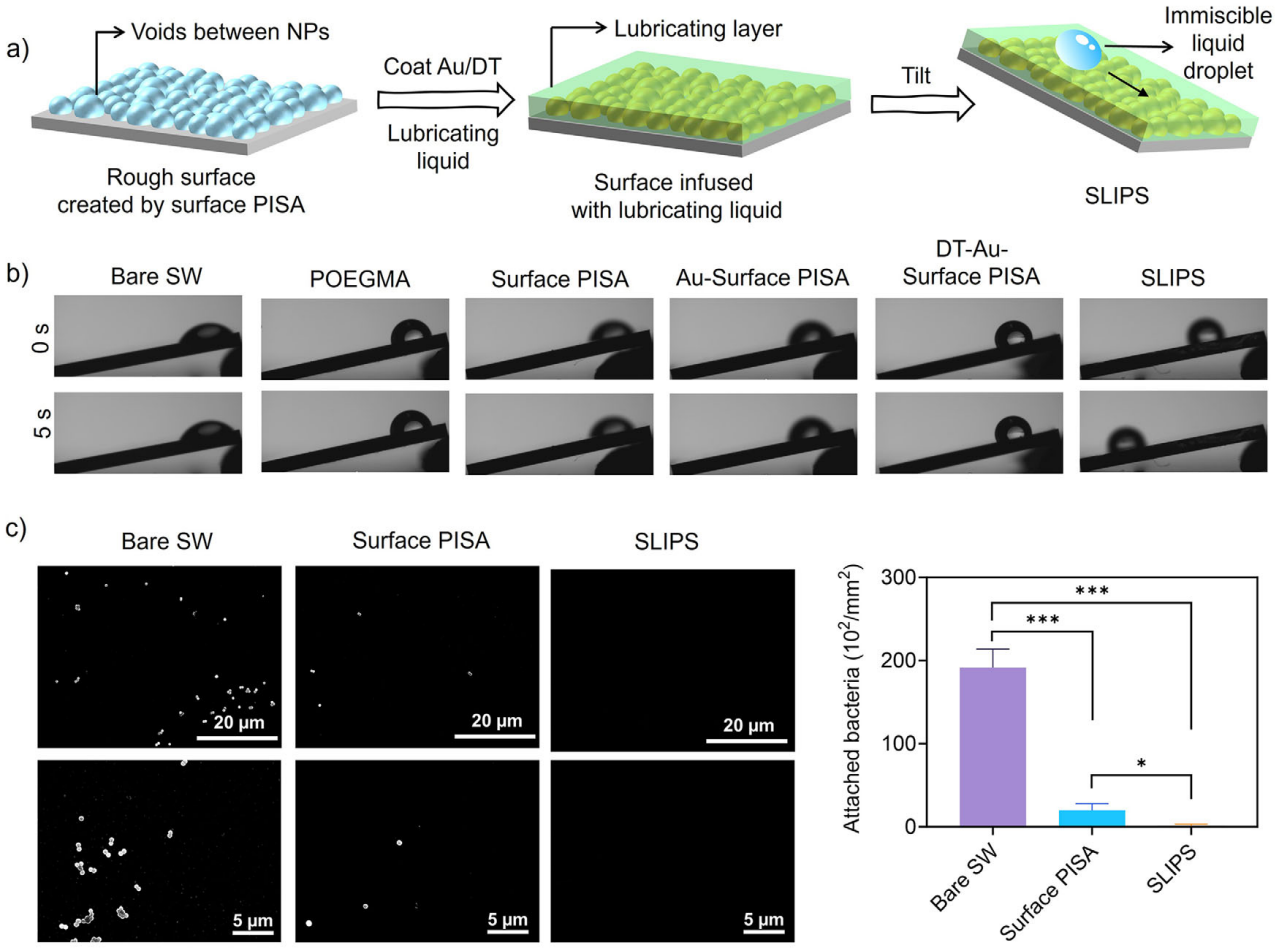
a) Schematic illustration of SLIPS enabled by surface PISA. b) Images of water droplets sliding off different surfaces at a tilt angle of 10^°^ at 0 and 5 s. c) SEM images (scale bar: top panel 20 µm, bottom panel: 5 µm) and the number of *S. aureus* adhered to different surfaces. ***P ≤ 0.001, *P ≤ 0.05, *n* = 3.

The use of surface PISA technology to develop innovative SLIPS^[^ ^78^, ^79^ ^]^ was also studied. SLIPS consist of a layer of lubricating liquids infused into micro‐ and nanostructured surfaces. The lubricating liquid forms a uniform and stable film, rendering the surface slippery and highly resistant to adhesion. Since its introduction by the group of Aizenberg in 2011,^[^ ^60^ ^]^ SLIPS has attracted significant attention and has found successful applications in various fields, including antifouling,^[^ ^80^, ^81^ ^]^ anti‐icing^[^ ^82^, ^83^ ^]^ and anti‐corrosion.^[^ ^84^, ^85^ ^]^ The key to creating SLIPS is developing micro‐ and nanostructured surfaces capable of generating sufficient capillary force to adsorb and retain the lubricating liquids. While several methods,^[^ ^86^ ^]^ such as lithography, layer‐by‐layer assembly and chemical vapour deposition, have been developed to fabricate micro‐ and nanostructured surfaces for SLIPS applications, we envisioned that surface PISA could offer a simpler alternative. The densely packed nanoparticles generated by surface PISA form voids and porous surface structures with a large surface area (Figure 7a), which may accommodate the infused lubricating liquids to develop SLIPS. We then demonstrated this proof of concept using wafers that had undergone surface PISA. Silicone oil was used as an example of a lubricating liquid. The hydrophilic POEGMA shells of the nanoparticles hindered silicone oil from directly infusing the voids and porous structures created by the surface PISA nanoparticles. Therefore, we coated the nanoparticle surface with a ∼5 nm gold (Au) layer, which was then modified with hydrophobic 1‐dodecanethiol (DT). These treatments preserved the rough surface features (Figure S23) and increased surface hydrophobicity (Figure S24), allowing silicone oil to adhere and permeate the surface. The silicone oil was infused into the surface by spin coating, forming a film with a thickness of approximately 680 nm and a contact angle of 106° (Figure S24). We then tested the slipperiness of the silicone oil‐infused surface and compared its performance to other surfaces with different modifications. As shown in Figure 7b, water droplets readily slid off the oil‐infused surface at a small tilt angle of 10° with a velocity of 0.085 cm s^−1^ (Video S5), but the droplets did not slide off the other surfaces. Additional sliding tests with other liquids (e.g., Coca Cola, orange juice, milk and dimethylformamide [DMF]) showed the same phenomenon (Figures S25 and S26), confirming the successful fabrication of SLIPS based on the surface PISA technology. SLIPS have been proven effective in preventing biofouling.^[^ ^87^, ^88^, ^89^ ^]^ Therefore, we further evaluated the antifouling properties of the developed SLIPS by testing their ability to resist the adhesion of *S. aureus*. As shown in Figure 7c, the SLIPS demonstrated even greater antifouling performance than the surface PISA‐modified surface, with negligible adhesion of *S. aureus* observed. This result highlights the superior ability of SLIPS to resist bacterial attachment, offering enhanced surface protection against biofouling.

### Encapsulation of Antibiotics Using Surface PISA

**Figure 8:**
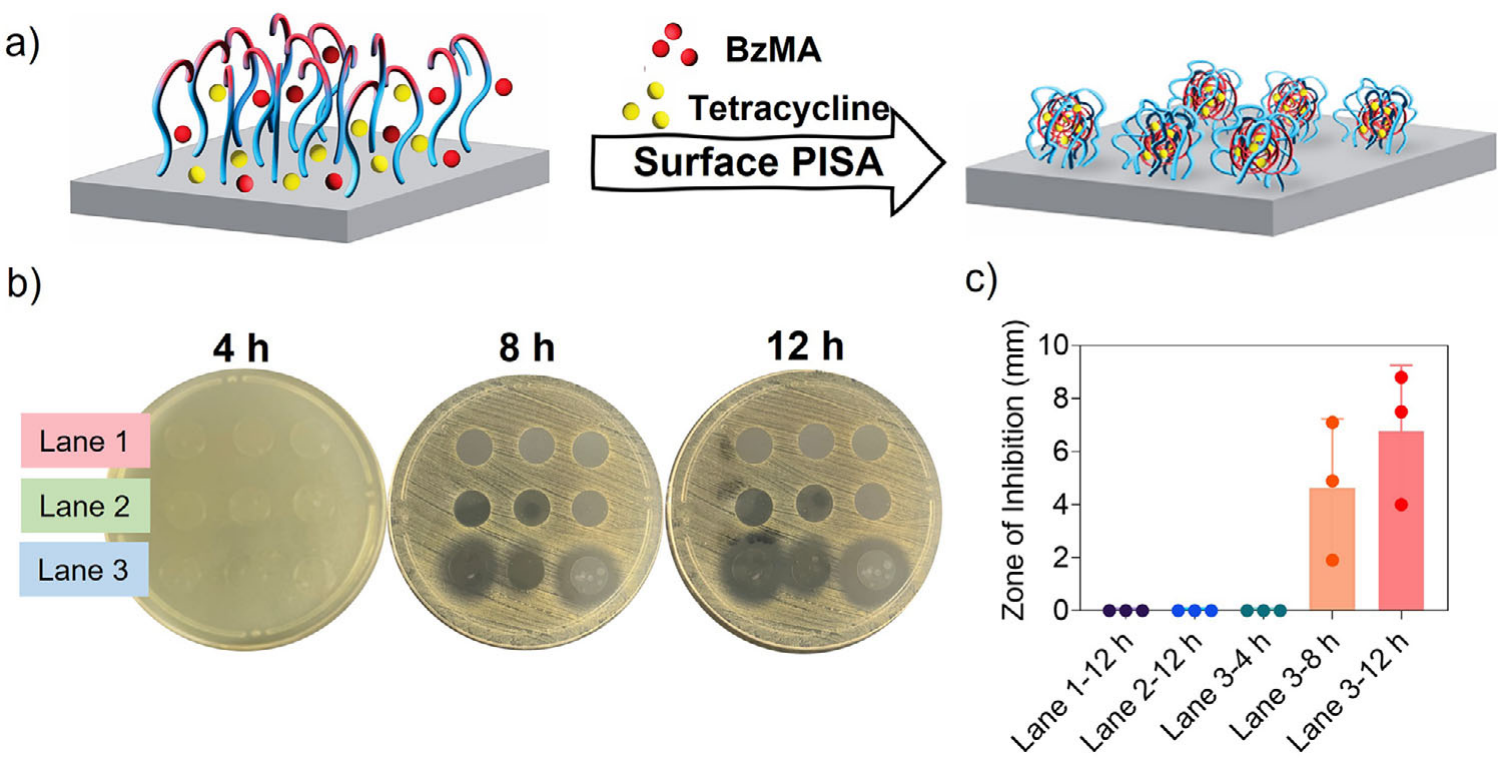
a) Schematic illustration of encapsulation of tetracycline via surface PISA. b) Zones of inhibition and c) quantitative analysis observed in a disc diffusion test against an *S. aureus* lawn with bare glass slips (Lane 1), surface PISA‐modified glass slips (Lane 2) and tetracycline‐encapsulated surface PISA‐modified glass slips (Lane 3).

Polymeric nanoparticles have been widely employed as drug delivery systems in a range of biomedical applications, including cancer therapy,^[^ ^90^ ^]^ vaccine delivery^[^ ^91^ ^]^ and antimicrobial treatments.^[^ ^92^ ^]^ The PISA process enables efficient encapsulation of functional molecules during the formation of polymeric nanoparticles and therefore has been increasingly applied in the field of drug delivery.^[^ ^93^, ^94^, ^95^ ^]^ While solution PISA has been extensively studied for drug encapsulation, encapsulating drugs via surface PISA has yet to be explored and can potentially lead to the development of local drug delivery systems. To demonstrate this concept, we conducted surface PISA to introduce antibiotics to the surface of glass slips to impart antimicrobial properties (Figure 8a). Tetracycline, a broad‐spectrum antibiotic, was used as a model antimicrobial agent for the encapsulation. During the in situ self‐assembly process, tetracycline is expected to be physically entrapped within the formed surface‐tethered nanoparticles. The growing PBzMA core provides a solvophobic environment that facilitates encapsulation through π–π stacking and hydrophobic interactions with tetracycline. As shown in Figure S27, nanoparticles were successfully formed on the surface of the glass slips via surface PISA in the presence of tetracycline. The addition of tetracycline to surface PISA did not alter the size, morphology and roughness of the surface nanoparticles. To evaluate the antimicrobial activity, the modified glass slips were placed on an agar plate which was uniformly inoculated with an *S. aureus* lawn (Figure 8b). The encapsulated tetracycline is expected to be released into the agar medium via diffusion from the surface‐tethered nanoparticles, thereby exerting an antibacterial effect. This effect was investigated by measuring the zone of inhibition around the glass slips over time and comparing the results to those from unmodified glass slips and surface PISA‐modified glass slips without tetracycline loading (Figure 8c). The results showed that neither the unmodified bare glass slips (Lane 1) nor those coated with empty surface PISA nanoparticles (Lane 2) exhibited antimicrobial activity, evidenced by the absence of inhibition zones around these samples. In contrast, the tetracycline‐loaded, surface PISA‐modified glass slips (Lane 3) displayed a time‐dependent increase in the zone of inhibition, suggesting that tetracycline was gradually released over time from the surface‐tethered nanoparticles. Notably, at the 4 h time point, no visible inhibition zone was observed. This delay in observable antimicrobial activity can be attributed to the gradual accumulation of tetracycline released from the surface nanoparticles into the agar medium, which had not yet reached an effective antibacterial concentration, as well as the early exponential growth phase of *S. aureus*, during which bacterial density remains low and inhibition is not yet macroscopically apparent. By 8 h, a distinct inhibition zone had formed, indicating that sufficient tetracycline had diffused from the surface to inhibit bacterial proliferation. After 12 h of incubation, the inhibition zone further expanded, suggesting sustained release of tetracycline from the surface and continued antimicrobial activity.

## Conclusion

In summary, we have expanded the scope of PISA, a well‐established and widely applied technology for the efficient preparation of polymeric nanoparticles with various morphologies, from a process predominately occurring in solution to one that can be applied to planar surfaces. By leveraging photopolymerisation technology, we systematically investigated the effect of several important parameters on the formation of nanoparticles on planar surfaces via the surface PISA approach. We found that the chain extension time, grafting density and length of surface‐tethered macro‐stabilisers, and the concentration of the core‐forming monomer all impact the size, density and surface roughness of the nanoparticles formed via surface PISA. Through DPD simulations, we gained in‐depth insights into the dynamic process of surface PISA as well as the effects of these critical parameters. We demonstrated that surface PISA provides a novel approach for creating nanoscale surface topography and modulating material‐bio interactions. Significantly, surface PISA enhances antifouling properties by more effectively reducing bacterial adhesion compared to conventional surface polymer brush technology. Furthermore, we showed that surface PISA offers a simple approach to developing rough surfaces with micro‐ and nanostructures, enabling the creation of innovative SLIPS with exceptional antifouling performance. Finally, we demonstrated that antibiotics can be readily introduced to material surfaces via the surface PISA approach, endowing the surfaces with antimicrobial properties. We believe this work is a significant step forward for PISA technology and will provide new opportunities for its broader applications.

## Conflict of Interests

The authors declare no conflict of interest.

## Supporting information



Supporting information

Supporting information

Supporting information

Supporting information

Supporting information

Supporting information

## Data Availability

The data that support the findings of this study are available from the corresponding author upon reasonable request.

## References

[1] N. J. Penfold , J. Yeow , C. Boyer , S. P. Armes , ACS Macro Lett. 2019, 8, 1029–1054. 35619484 10.1021/acsmacrolett.9b00464

[2] N. J. Warren , S. P. Armes , J. Am. Chem. Soc. 2014, 136, 10174–10185. 24968281 10.1021/ja502843f PMC4111214

[3] J. Tan , H. Sun , M. Yu , B. S. Sumerlin , L. Zhang , ACS Macro Lett. 2015, 4, 1249–1253. 35614822 10.1021/acsmacrolett.5b00748

[4] J. Wan , B. Fan , S. H. Thang , Chem. Sci. 2022, 13, 4192–4224. 35509470 10.1039/d2sc00762b PMC9006902

[5] C. Liu , C.‐Y. Hong , C.‐Y. Pan , Polym. Chem. 2020, 11, 3673–3689.

[6] S. Zhang , R. Li , Z. An , Angew. Chem. Int. Ed. 2024, 63, e202315849. 10.1002/anie.202315849 38155097

[7] J. Yeow , C. Boyer , Adv. Sci. 2017, 4, 1700137. 10.1002/advs.201700137 PMC5514979 28725534

[8] E. Jones , M. Semsarilar , P. Wyman , M. Boerakker , S. Armes , Polym. Chem. 2016, 7, 851–859. 10.1021/acs.macromol.5b02385 PMC4745608 26893528

[9] C. György , S. P. Armes , Angew. Chem. Int. Ed. 2023, 62, e202308372. 10.1002/anie.202308372 PMC10952376 37409380

[10] X. Wang , C. A. Figg , X. Lv , Y. Yang , B. S. Sumerlin , Z. An , ACS Macro Lett. 2017, 6, 337–342 35610849 10.1021/acsmacrolett.7b00099

[11] J. Lesage de la Haye , X. Zhang , I. Chaduc , F. Brunel , M. Lansalot , F. d'Agosto , Angew. Chem. Int. Ed. 2016, 55, 3739–3743. 10.1002/anie.201511159 26880016

[12] Z. Zhao , S. Lei , M. Zeng , M. Huo , Aggregate 2024, 5, e418.

[13] Q. Gu , H. Li , E. J. Cornel , J. Du , Cell Rep. Phys. Sci. 2023, 4, 101495.

[14] X. Luo , S. Zhao , Y. Chen , L. Zhang , J. Tan , Macromolecules 2021, 54, 2948–2959.

[15] G. Li , C. Duclos , R. G. Ricarte , Soft Matter 2024, 20, 7214–7226. 39224056 10.1039/d4sm00654b

[16] C. Cao , F. Chen , C. J. Garvey , M. H. Stenzel , ACS Appl. Mater. Interfaces 2020, 12, 30221–30233. 32515935 10.1021/acsami.0c09054

[17] A. B. Korpusik , Y. Tan , J. B. Garrison , W. Tan , B. S. Sumerlin , Macromolecules 2021, 54, 7354–7363.

[18] J.‐M. Noy , F. Chen , D. T. Akhter , Z. H. Houston , N. L. Fletcher , K. J. Thurecht , M. H. Stenzel , Biomacromolecules 2020, 21, 2320–2333. 32343128 10.1021/acs.biomac.0c00257

[19] H. Sun , W. Cao , N. Zang , T. D. Clemons , G. M. Scheutz , Z. Hu , M. P. Thompson , Y. Liang , M. Vratsanos , X. Zhou , Angew. Chem. 2020, 132, 19298–19304. 10.1002/anie.202006385 PMC7722202 32659039

[20] X. Liu , M. Sun , J. Sun , J. Hu , Z. Wang , J. Guo , W. Gao , J. Am. Chem. Soc. 2018, 140, 10435–10438. 30084632 10.1021/jacs.8b06013

[21] C. Ma , X. Liu , G. Wu , P. Zhou , Y. Zhou , L. Wang , X. Huang , ACS Macro Lett. 2017, 6, 689–694. 35650871 10.1021/acsmacrolett.7b00422

[22] D. J. Rucco , B. E. Barnes , J. B. Garrison , B. S. Sumerlin , D. A. Savin , Biomacromolecules 2020, 21, 5077–5085. 33169973 10.1021/acs.biomac.0c01225

[23] Y. Chang , X. Xu , R. Zhang , H. Peng , K. Liu , A. K. Whittaker , C. Fu , Macromolecules 2023, 57, 263–271.

[24] J. Chang , H. Zhou , C. Li , J. Sun , Q. Wang , Y. Li , W. Zhao , Biomacromolecules 2023, 24, 2918–2927. 37235210 10.1021/acs.biomac.3c00291

[25] V. M. Panakkal , D. Havlicek , E. Pavlova , M. Filipová , S. Bener , D. Jirak , O. Sedlacek , Biomacromolecules 2022, 23, 4814–4824. 36251480 10.1021/acs.biomac.2c00981 PMC10797588

[26] W. Zhao , H. T. Ta , C. Zhang , A. K. Whittaker , Biomacromolecules 2017, 18, 1145–1156. 28339189 10.1021/acs.biomac.6b01788

[27] A. Belluati , S. Jimaja , R. J. Chadwick , C. Glynn , M. Chami , D. Happel , C. Guo , H. Kolmar , N. Bruns , Nat. Chem. 2024, 16, 564–574. 38049652 10.1038/s41557-023-01391-y PMC10997521

[28] B. P. Bastakoti , J. Perez‐Mercader , Angew. Chem. Int. Ed. 2017, 56, 12086–12091. 10.1002/anie.201703816 28643367

[29] P. Shi , Y. Qu , C. Liu , H. Khan , P. Sun , W. Zhang , ACS Macro Lett. 2016, 5, 88–93. 35668584 10.1021/acsmacrolett.5b00928

[30] L. Fan , Z. Zeng , R. Zhu , A. Liu , H. Che , M. Huo , Chem. Mat. 2022, 34, 6408–6419.

[31] M. Zhang , S. Gong , K. Hakobyan , Z. Gao , Z. Shao , S. Peng , S. Wu , X. Hao , Z. Jiang , E. H. Wong , Adv. Sci. 2024, 11, 2309006. 10.1002/advs.202309006 PMC10870077 38072658

[32] Y. T. Cheng , Q. Xia , H. Liu , M. B. Solomon , E. R. Brisson , L. D. Blackman , C. D. Ling , M. Müllner , ACS Appl. Mater. Interfaces 2023, 15, 12261–12272. 36821625 10.1021/acsami.2c18928

[33] Z. Liu , W. Li , W. Sheng , S. Liu , R. Li , Q. Li , D. Li , S. Yu , M. Li , Y. Li , J. Am. Chem. Soc. 2023, 145, 5310–5319. 36758639 10.1021/jacs.2c12977

[34] W. Hou , H. Wang , Y. Cui , Y. Liu , X. Ma , H. Zhao , Macromolecules 2019, 52, 8404–8414.

[35] Y. Zheng , Y. Huang , Z. M. Abbas , B. C. Benicewicz , Polym. Chem. 2017, 8, 370–374.

[36] B. Niu , H. Huang , L. Luo , L. Zhang , J. Tan , Chin. Chem. Lett. 2025, 36, 110431.

[37] W. Zhong , W. Hou , Y. Liu , L. Liu , H. Zhao , Langmuir 2020, 36, 12649–12657. 33070609 10.1021/acs.langmuir.0c02201

[38] B. Niu , H. Huang , L. Zhang , J. Tan , ACS Macro Lett. 2024, 13, 577–585. 38648524 10.1021/acsmacrolett.4c00098

[39] M. S. Lord , M. Foss , F. Besenbacher , Nano Today 2010, 5, 66–78.

[40] X. Yao , R. Peng , J. Ding , Adv. Mater. 2013, 25, 5257–5286. 24038153 10.1002/adma.201301762

[41] R. J. Crawford , H. K. Webb , V. K. Truong , J. Hasan , E. P. Ivanova , Adv. Colloid Interface Sci. 2012, 179‐182, 142–149. 10.1016/j.cis.2012.06.015 22841530

[42] M. Nikkhah , F. Edalat , S. Manoucheri , A. Khademhosseini , Biomaterials 2012, 33, 5230–5246. 22521491 10.1016/j.biomaterials.2012.03.079 PMC3619386

[43] K. Modaresifar , S. Azizian , M. Ganjian , L. E. Fratila‐Apachitei , A. A. Zadpoor , Acta Biomater. 2019, 83, 29–36. 30273746 10.1016/j.actbio.2018.09.059

[44] H. Durand , A. Whiteley , P. Mailley , G. Nonglaton , ACS Appl. Bio Mater. 2022, 5, 4718–4740. 10.1021/acsabm.2c00586 36162127

[45] C. Mas‐Moruno , B. Su , M. J. Dalby , Adv. Healthcare Mater. 2019, 8, 1801103. 10.1002/adhm.201801103 30468010

[46] D. Li , Q. Zheng , Y. Wang , H. Chen , Polym. Chem. 2014, 5, 14–24.

[47] P. Decuzzi , M. Ferrari , Biomaterials 2010, 31, 173–179. 19783034 10.1016/j.biomaterials.2009.09.018

[48] L. Zhao , R. Chen , L. Lou , X. Jing , Q. Liu , J. Liu , J. Yu , P. Liu , J. Wang , Appl. Surf. Sci. 2020, 511, 145564.

[49] Y. Lee , Y.‐W. Chung , J. Park , K. Park , Y. Seo , S.‐N. Hong , S. H. Lee , H. Jeon , J. Seo , Sci. Rep. 2020, 10, 17454. 33060752 10.1038/s41598-020-74517-8 PMC7566624

[50] X. Chen , X. He , X. Suo , J. Huang , Y. Gong , Y. Liu , H. Li , Appl. Surf. Sci. 2016, 388, 385–391.

[51] X. Xu , Y. Chang , Y. Gong , Y. Zhang , Y. Yu , H. Peng , C. Fu , ACS Appl. Polym. Mater. 2023, 6, 1–27.

[52] W. J. Yang , K.‐G. Neoh , E.‐T. Kang , S. L.‐M. Teo , D. Rittschof , Prog. Polym. Sci. 2014, 39, 1017–1042.

[53] C. Feng , X. Huang , Acc. Chem. Res. 2018, 51, 2314–2323. 30137964 10.1021/acs.accounts.8b00307

[54] M. Fromel , E. M. Benetti , C. W. Pester , ACS Macro Lett. 2022, 11, 415–421. 35575317 10.1021/acsmacrolett.2c00114

[55] B. Li , B. Yu , Q. Ye , F. Zhou , Acc. Chem. Res. 2015, 48, 229–237. 25521476 10.1021/ar500323p

[56] R. Wang , Q. Wei , W. Sheng , B. Yu , F. Zhou , B. Li , Angew. Chem. 2023, 135, e202219312. 10.1002/anie.202219312 36950880

[57] W.‐L. Chen , R. Cordero , H. Tran , C. K. Ober , Macromolecules 2017, 50, 4089–4113.

[58] A. M. Bhayo , Y. Yang , X. He , Prog. Mater. Sci. 2022, 130, 101000.

[59] M. Li , M. Fromel , D. Ranaweera , S. Rocha , C. Boyer , C. W. Pester , ACS Macro Lett. 2019, 8, 374–380. 35651140 10.1021/acsmacrolett.9b00089

[60] T.‐S. Wong , S. H. Kang , S. K. Tang , E. J. Smythe , B. D. Hatton , A. Grinthal , J. Aizenberg , Nature 2011, 477, 443–447. 21938066 10.1038/nature10447

[61] Y. Zhang , Z. Wang , K. Matyjaszewski , J. Pietrasik , Macromol. Rapid Commun. 2019, 40, 1800331. 10.1002/marc.201800331 29974536

[62] E. T. Garrett , Y. Pei , A. B. Lowe , Polym. Chem. 2016, 7, 297–301.

[63] J. Yeow , J. Xu , C. Boyer , ACS Macro Lett. 2015, 4, 984–990. 35596469 10.1021/acsmacrolett.5b00523

[64] J. Yeow , S. Shanmugam , N. Corrigan , R. P. Kuchel , J. Xu , C. Boyer , Macromolecules 2016, 49, 7277–7285.

[65] J. Xu , S. Shanmugam , H. T. Duong , C. Boyer , Polym. Chem. 2015, 6, 5615–5624.

[66] C. Fu , B. Demir , S. Alcantara , V. Kumar , F. Han , H. G. Kelly , X. Tan , Y. Yu , W. Xu , J. Zhao , C. Zhang , H. Peng , C. Boyer , T. Woodruff , S. Kent , D. Searles , A. K. Whittaker , Angew. Chem. Int. Ed. 2020, 59, 4729–4735. 10.1002/anie.201914119 31951063

[67] Y. Jiang , N. Xu , J. Han , Q. Yu , L. Guo , P. Gao , X. Lu , Y. Cai , Polym. Chem. 2015, 6, 4955–4965.

[68] Q. Qu , G. Liu , X. Lv , B. Zhang , Z. An , ACS Macro Lett. 2016, 5, 316–320. 35614727 10.1021/acsmacrolett.6b00066

[69] Y. Liu , Y. Li , J. He , K. J. Duelge , Z. Lu , Z. Nie , J. Am. Chem. Soc. 2014, 136, 2602–2610. 24447129 10.1021/ja412172f

[70] K. Zhang , H.‐M. Gao , D. Xu , Z.‐Y. Lu , Soft Matter 2019, 15, 890–900. 30633294 10.1039/c8sm02472c

[71] X. Su , M. Yang , D. Hao , X. Guo , L. Jiang , J. Colloid Interface Sci. 2021, 598, 104–112. 33895532 10.1016/j.jcis.2021.04.031

[72] A. Francone , S. Merino , A. Retolaza , J. Ramiro , S. A. Alves , J. V. de Castro , N. M. Neves , A. Arana , J. M. Marimon , C. M. S. Torres , Surf. Interfaces 2021, 27, 101494. 34957348 10.1016/j.surfin.2021.101494 PMC8500737

[73] M. Mu , S. Liu , W. DeFlorio , L. Hao , X. Wang , K. S. Salazar , M. Taylor , A. Castillo , L. Cisneros‐Zevallos , J. K. Oh , Langmuir 2023, 39, 5426–5439. 37014907 10.1021/acs.langmuir.3c00091 PMC10848269

[74] L. Li , B. Yan , L. Zhang , Y. Tian , H. Zeng , Chem. Commun. 2015, 51, 15780–15783. 10.1039/c5cc06852e 26364998

[75] T. Kang , X. Banquy , J. Heo , C. Lim , N. A. Lynd , P. Lundberg , D. X. Oh , H.‐K. Lee , Y.‐K. Hong , D. S. Hwang , ACS Nano 2016, 10, 930–937. 26695175 10.1021/acsnano.5b06066 PMC4932843

[76] E. Shin , C. Lim , U. J. Kang , M. Kim , J. Park , D. Kim , W. Choi , J. Hong , C. Baig , D. W. Lee , Macromolecules 2020, 53, 3551–3562.

[77] G. Morgese , L. Trachsel , M. Romio , M. Divandari , S. N. Ramakrishna , E. M. Benetti , Angew. Chem. 2016, 128, 15812–15817. 10.1002/anie.201607309 27775203

[78] W. Yan , S. Xue , B. Xiang , X. Zhao , W. Zhang , P. Mu , J. Li , Chem. Commun. 2023, 59, 2182–2198. 10.1039/d2cc06688b 36723187

[79] Y. Jia , Y. Yang , X. Cai , H. Zhang , ACS Biomater. Sci. Eng. 2024, 10, 3655–3672. 38743527 10.1021/acsbiomaterials.4c00422

[80] L. Xiao , J. Li , S. Mieszkin , A. Di Fino , A. S. Clare , M. E. Callow , J. A. Callow , M. Grunze , A. Rosenhahn , P. A. Levkin , ACS Appl. Mater. Interfaces 2013, 5, 10074–10080. 24067279 10.1021/am402635p

[81] Z. Tong , F. Gao , S. Chen , L. Song , J. Hu , Y. Hou , J. Lu , M. K. Leung , X. Zhan , Q. Zhang , Adv. Mater. 2024, 36, 2308972. 10.1002/adma.202308972 37917884

[82] Y. Yuan , H. Xiang , G. Liu , L. Wang , H. Liu , R. Liao , Adv. Mater. Interfaces 2022, 9, 2101968.

[83] C. Liu , Y. Li , C. Lu , Y. Liu , S. Feng , Y. Liu , ACS Appl. Mater. Interfaces 2020, 12, 25471–25477. 32379411 10.1021/acsami.0c05954

[84] T. Xiang , M. Zhang , H. R. Sadig , Z. Li , M. Zhang , C. Dong , L. Yang , W. Chan , C. Li , Chem. Eng. J. 2018, 345, 147–155.

[85] S. Li , F. Zhao , Y. Bai , Z. Ye , Z. Feng , X. Liu , S. Gao , X. Pang , M. Sun , J. Zhang , Chem. Eng. J. 2022, 431, 133945.

[86] L. Xia , S. Zhang , Z. Guo , Adv. Mater. Interfaces 2023, 10, 2202212.

[87] Z. Tong , L. Song , S. Chen , J. Hu , Y. Hou , Q. Liu , Y. Ren , X. Zhan , Q. Zhang , Adv. Funct. Mater. 2022, 32, 2201290.

[88] Y. Jing , F. Meng , F. Wang , L. Liu , ACS Appl. Mater. Interfaces 2025, 17, 4009–4021. 39746879 10.1021/acsami.4c19298

[89] P. Wang , D. Zhang , S. Sun , T. Li , Y. Sun , ACS Appl. Mater. Interfaces 2017, 9, 972–982. 27992173 10.1021/acsami.6b09117

[90] S. Parveen , S. K. Sahoo , J. Drug Target. 2008, 16, 108–123. 18274932 10.1080/10611860701794353

[91] D. Wibowo , S. H. Jorritsma , Z. J. Gonzaga , B. Evert , S. Chen , B. H. Rehm , Biomaterials 2021, 268, 120597. 33360074 10.1016/j.biomaterials.2020.120597 PMC7834201

[92] S. J. Lam , E. H. Wong , C. Boyer , G. G. Qiao , Prog. Polym. Sci. 2018, 76, 40–64.

[93] M. Lages , J. Nicolas , Prog. Polym. Sci. 2023, 137, 101637.

[94] H. Phan , V. Taresco , J. Penelle , B. Couturaud , Biomater. Sci. 2021, 9, 38–50. 33179646 10.1039/d0bm01406k

[95] E. G. Hochreiner , B. G. van Ravensteijn , J. Polym. Sci. 2023, 61, 3186–3210.

