# Supplementary material for: Polymerisation‐Induced Self‐Assembly on Planar Surfaces: A New Approach for Controlling Surface Topography and Modulating Material‐Bio Interactions
Source: Angew Chem Int Ed Engl. 2025 Aug 14;64(39):e202507194. doi: 10.1002/anie.202507194 (PMC12455395; doi:10.1002/anie.202507194)
Supplement: Supplementary file 1 — Supporting information [file ANIE-64-e202507194-s003.docx]

**Polymerisation-Induced Self-Assembly on Planar Surfaces: A New Approach for Controlling Surface Topography and Modulating Material-Bio Interactions**

Xin Xu,^a^ Jia-Qi Xu,^b^ You-Liang Zhu,^b^ Yixin Chang,^a^ Yuhao Zhang,^a^ Hui Peng,^a^ Zhong-Yuan Lu,^b*^ Andrew Whittaker,^a,c*^ Changkui Fu^a*^

^a^Australian Institute for Bioengineering and Nanotechnology, The University of Queensland, St Lucia, Queensland 4072, Australia

^b^State Key Laboratory of Supramolecular Structure and Materials, College of Chemistry, Jilin University, Changchun 130012, China

^c^Australian Research Council Centre of Excellence for Green Electrochemical Transformation of Carbon Dioxide, The University of Queensland, St. Lucia, Queensland 4072, Australia.

Email: [luzhy@jlu.edu.cn](mailto:luzhy@jlu.edu.cn); [a.whittaker@uq.edu.au](mailto:a.whittaker@uq.edu.au); [changkui.fu@uq.edu.au](mailto:changkui.fu@uq.edu.au)

**Experimental Section**

**Materials**

Sodium hydroxide mini pellets (NaOH, 99%) were purchased from Chem-Supply. 1-ethyl-3-(3-dimethylaminopropyl) carbodiimide hydrochloride (EDC·HCl) was purchased from Thermo Fisher Scientific. 3-aminopropyl triethoxysilane (APTES, 99%), 3-(triethoxysilyl)propionitrile (TESPN, 97%), N-hydroxysuccinimide (NHS, 98%), oligo(ethylene glycol) methyl ether methacrylate (OEGMA, average *M*_n_ = 300), benzyl methacrylate (BzMA, 96%), 4-cyano-4-[(dodecylsulfanylthiocarbonyl)sulfanyl]pentanoic acid (CDTPA, 97%), and Eosin Y disodium salt (C_20_H_6_Br_4_Na_2_O_5_, dye content ≥85%), tetracycline (98.0 - 102.0%), silicone oil (viscosity: 332-368 cP) were purchased from Sigma-Aldrich and used as received. Staphylococcus aureus (ATCC 6538) was purchased from American Type Culture Collection (ATCC).

**X-ray Photoelectron Spectroscopy (XPS)**

The XPS data was acquired on a Kratos Axis Supra Plus XPS equipped with a dual monochromated Al Kα/Ag Lα X-ray source. The composition and chemical bond state of C 1s, N 1s, O 1s, S 2p, Si 2s, and Si 2p were detected. Casa XPS software was used to collect and analyse the data.

**Atomic Force Microscope (AFM)**

The surface morphology was analysed using a Bruker Dimension XR atomic force microscope (AFM) operating in ScanAsyst-air mode. A ScanAsyst-air cantilever, with a resonance frequency of 70 kHz, a spring constant of 0.4 N/m, and a tip radius of less than 12 nm, was employed to capture height images at a scan rate of 1 Hz.

**Nuclear Magnetic Resonance (NMR)**

^1^H NMR spectra were obtained using the Bruker Avance 500 MHz high-resolution NMR spectrometer at 298 K. All chemical shifts are reported in ppm (δ).

**Size-Exclusion Chromatography (SEC)**

The molecular weight (*M*_n_) and molecular weight dispersity (*Đ* = *M*_w_/*M*_n_) of the polymers were determined by SEC using a Waters Alliance 2690 system with a 2414 RI detector, 2489 UV/Visible detector, 717 Plus Autosampler, and 1515 Isocratic HPLC pump. THF was the mobile phase at 1 mL/min. System calibration was performed with polystyrene standards (6.82 × 10^2^ to 1.67 × 10^6^ g/mol).

**Contact Angle Measurements**

Surface hydrophilicity was evaluated using water contact angles measured with the tangent fitting algorithm. Each dry sample received 3 μL of deionised water dispensed via an automatic controller. Testing was conducted four times at various positions on the surface.

**Thickness Measurements by Ellipsometry**

The layer thickness was measured using a Woollam VUV-VASE32 variable-angle spectroscopic ellipsometer employing the Cauchy model. Each sample underwent four scans at angles ranging from 45 to 65° with 5° intervals across wavelengths from 245 to 1000 nm. The acquired data were analysed using CompleteEASE software for fitting.

**Grafting Density Determination**

The grafting density of the polymer brushes on the surfaces was determined using a method described in previous literature.^[1]^ The bulk density of POEGMA was taken as 1.19 g/cm³.

**Dissipative Particle Dynamics Simulation Method**

Dissipative particle dynamics (DPD) is an effective mesoscale simulation method that is suitable to investigate the self-assembly morphology of diblock copolymers.^[2]^ The motion of coarse-grained particles (composed of a group of atoms or molecules) over time follows Newton’s equation of motion:

$mdv_{i}/dt=f_{i},$ (1)

where the force $f_{i}$ on the particle $i$ contains three parts, including conservative force $F^{C}$, dissipative force $F^{D}$, and random force $F^{R}$:

$f_{i}=\sum_{j\neq i} (F_{ij}^{C}+F_{ij}^{D}+F_{ij}^{R})$. (2)

The $f_{i}$ is summed over all the inter-bead forces between particle $i$ and particle $j$ within the cutoff radius $r_{c}$. The conservative force is weakly repulsive and is given by

$F_{ij}^{C}=\left\{ \begin{matrix} a_{ij}(1-r_{ij})\hat{r}_{ij} & (r_{ij}<1) \\ 0 & (r_{ij}\geq1) \end{matrix} \right.$ , (3)

where $a_{ij}$ measures the maximum repulsion between beads $i$ and $j$, $r_{ij}=\left| r_{i}-r_{j} \right|/r_{c}$, and $\hat{r}_{ij}=(r_{i}-r_{j})/\left| r_{i}-r_{j} \right|$. The dissipative force and random force are given by

$F_{ij}^{D}=-\gamma\omega^{D}(r_{ij})(\hat{r}_{ij}\cdot v_{ij})\hat{r}_{ij}$, (4)

and

$F_{ij}^{R}=\sigma\omega^{R}(r_{ij})\theta_{ij}\hat{r}_{ij}$, (5)

respectively. $\gamma$ is a simulation parameter related to viscosity arising from the interactions between beads, and $v_{ij}=v_{i}-v_{j}$. $\theta_{ij}$ is a zero-mean Gaussian random variable of unit variance, and $\sigma^{2}=2k_{B}T\gamma$, where $k_{B}$ is the Boltzmann constant and $T$ is the temperature. The $\omega^{D}$ and $\omega^{R}$ is given by

$\omega^{D}(r_{ij})=[\omega^{R}(r_{ij})]^{2}=\left\{ \begin{matrix} (1-r_{ij})^{2} & (r_{ij}<1) \\ 0 & (r_{ij}\geq1) \end{matrix} \right.$ (6)

In the bead-spring model, adjacent particles are connected by harmonic springs. The spring force $F^{S}$ is given by

$F_{ij}^{S}=-k(r_{ij}-r_{s})\hat{r}_{ij}$ (7)

**Coarse-grained model**

In coarse-grained (CG) model, a layer of W type beads arranged in a hexagonal pattern represent the surface of silicon wafer (Figure S15). The W type beads are immobile and interacted with other types of CG beads with Weeks-Chandler-Andersen (WCA) potential.^[3]^ As shown in Figure S15, a monomeric unit of OEGMA and BzMA is coarse-grained into three beads and two beads, respectively. Two ethanol molecules are taken into one bead (type C). In DPD simulations, the reduced units of energy, length and mass are *ε_0_ = k_B_T*, *σ_0_=*1 nm, and *m_0_* = amu, respectively. The pairwise interaction parameters $a_{ij}$ are calculated from the Flory-Huggins parameters χ with $\alpha_{ij}=25+3.27\chi_{ij}$. The Flory-Huggins parameters are estimated from the solubility parameter δ of the species by $\chi_{ij}=V_{r}{(\delta_{i}-\delta_{j})}^{2}/RT$, where $V_{r}=min(V_{i}, V_{j})$ and R is gas constant. The solubility parameters, Flory-Huggins parameters, and calculated interaction parameters are given below.^[4]^ The harmonic spring interactions employ the parameters *k* = 4.0 and $r_{s}$=0.0. The surface-RAFT process is simulated by a polymerisation model which was developed by us previously.^[5]^ The polymerisation model employed a cut off radius of 1.0, a reaction period of 100 time steps, and a reaction probability of 0.02, by referring to our previous simulations.^[6]^ A trajectory of 3 million time steps are integrated with a time step of 0.005. Four grafting densities including 0.01, 0.05, 0.10, and 0.15 chains/nm^2^ with randomly distributed grafting roots are considered in our simulations. All simulations were performed on an in-house developed simulation package.^[7]^


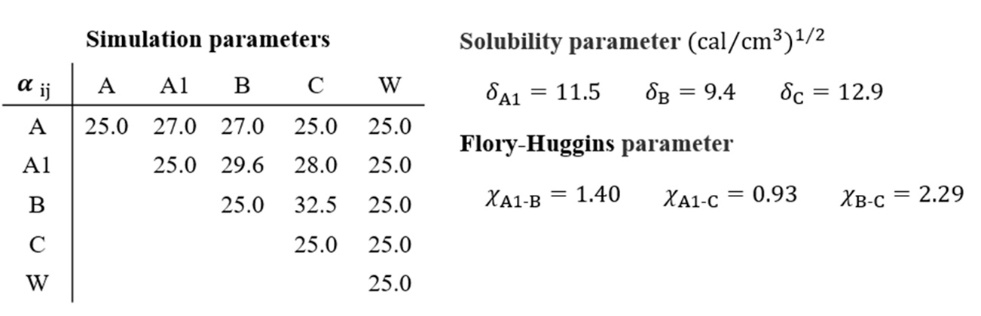


**Synthesis of Surface-CDTPA**

Square silicon wafers (10 mm diameter, 1 mm thickness) or round microscope glass coverslips (12 mm diameter, 0.1 mm thickness) were used as the planar surface substrates for further modification. Cleaned silicon wafers or glass slips were treated with 1 M sodium hydroxide solution in Milli-Q water for 3 h followed by water rinse until the pH reached 7. Afterward, the surfaces were rinsed consecutively with isopropanol, ethanol, and toluene. Then, the surfaces were treated with 10% solution of a mixture of APTES/TESPN (10%:90%, 50%:50%, or 100%/0%) in toluene for 1 h at room temperature followed by washing with toluene, then treated with 0.1 M citric acid for 5 min, and finally washed with water. The synthesis of CDTPA-NHS was based on previously reported protocols.^[8]^ In short, NHS (0.74 g, 6.44 mmol) and EDC·HCl (1.23 g, 6.44 mmol) were added to the solution of CDTPA (2 g, 4.95 mmol) in 50 mL dry DCM under an ice bath. The mixture was then stirred for 1 h at 0 °C and then for 9 h at room temperature followed by washing with saturated aqueous NaHCO_3_, drying over anhydrous Na_2_SO_4_. The filtered solution was evaporated to remove the solvent and dried under vacuum to yield CDTPA-NHS. Each surface was immersed in a solution of CDTPA-NHS (1.5 mg, 0.0028 mmol) in DMF (1.5 mL) with shaking for 48 h at room temperature. After reaction, the surfaces were sequentially washed with DMF, methanol, and acetone, and dried under vacuum at room temperature for subsequent uses.

**Synthesis of Surface-tethered Macro-Stabilisers (Surface-POEGMA)**

CDTPA-functionalised silicon wafers or glass slips were immersed in a solution of OEGMA (70 mg, 0.233 mmol), CDTPA (1.88 mg, 0.0047 mmol), and Eosin Y disodium salt (0.03 mg, 0.00004 mmol) in DMSO (400 µL) in a 96 well plate. The polymerisations were conducted under irradiation with LED light (*λ*_m_ = 405 nm; maximal irradiance = 3.5 mW/cm^2^ at 5 cm distance) for 18 h at room temperature. Monomer conversion was determined by comparing the integral of protons of the vinyl group and methylene adjacent to the ester group of the monomer before and after polymerisation. The POEGMA-grafted surfaces were rinsed with DMSO, methanol and acetone, and dried under vacuum at 30 °C. The free polymers were collected from the reaction and purified by dialysis against water followed by lyophilisation for further NMR and SEC characterisation.

**Synthesis of Surface-Tethered Macro-Stabilisers (Surface-PDMA)**

The synthesis of surface-tethered PDMA macro-stabilisers followed a similar protocol to that of surface-POEGMA. Specifically, CDTPA-functionalised silicon wafers were placed in a 96-well plate and immersed in a solution containing DMA (70 mg, 0.706 mmol), CDTPA (5.7 mg, 0.014 mmol), and Eosin Y disodium salt (0.1 mg, 0.00014 mmol) in 400 µL of DMSO. The reaction mixture was irradiated with blue LED light irradiation and allowed to proceed at room temperature for 15 hours. The PDMA-grafted wafers were rinsed with DMSO, methanol and acetone, and dried under vacuum at 30 °C. The free PDMA was characterised by NMR and SEC. DP_NMR_=36, *M*_n, SEC_=4340 g/mol, PDI=1.01.

**Surface PISA with Monomer BzMA**

POEGMA-grafted surfaces were immersed in a solution containing BzMA (70 mg, 0.4 mmol), Eosin Y disodium salt (0.03 mg, 0.00004 mmol) in ethanol (400 µL) in a 96 well plate. The PISA process was conducted under LED light (*λ*_m_ = 405 nm; irradiance = 3.5 mW/cm^2^ at 5 cm distance) for varying durations of 12, 24, 48, 72 and 144 h at room temperature. After reaction, the surfaces were rinsed several times with ethanol and dried with nitrogen stream, followed by vacuum drying at room temperature.

All the surface PISA was conducted in a similar manner.

**Polymerisation Kinetics of Solution PISA**

POEGMA-CDTPA (150 mg, 0.012 mmol) and BzMA (1.04 g, 5.9 mmol) were dissolved in ethanol (6 mL), followed by adding Eosin Y disodium salt solution (0.98 mg, 0.0014 mmol). The mixture was then placed in an ice bath and degassed for 20 min. Subsequently, the polymerisation solution was conducted under irradiation of LED light (*λ*_m_ = 405 nm; irradiance = 3.5 mW/cm² at 5 cm distance). Samples were collected at specific time points and further analysed by ¹H NMR, SEC, and TEM.

**Bacterial Adhesion Assay**

The polymer-grafted silicon wafers were sterilised by immersing them in ethanol for 20 min and then dried using airflow. Then, the silicon wafers were placed in 24 well plate followed by the addition of 300 μL *S.aureus* aliquots (8 × 10^6^ CFU/mL). These were incubated for 10 h in a shaking incubator at 37 °C and 25 rpm. After incubation, the bacterial suspensions were removed, and the silicon wafers were gently washed with PBS. The bacteria on the wafers were then fixed with 2.5% glutaraldehyde in PBS and stored at 4°C overnight. After dehydration with ethanol and platinum coating, the attached bacteria on the surface were observed using a scanning electron microscope (SEM).

**Fabrication of Slippery Liquid-infused Surface (SLIPS) Based on Surface PISA**

High density surface PISA-modified silicon wafers were first coated with a 5 nm thick gold layer via an electron beam evaporator to ensure uniform coverage. The gold-coated wafers were then immersed in a 5% (v/v) 1-dodecanethiol solution in ethanol for 20 min, followed by thorough rinsing with ethanol and drying in an oven at 50 ºC for 24 h. To further create SLIPS, 40 µL of silicone oil was deposited onto the functionalised wafers and evenly distributed via spin coating at 5000 rpm for 40 s. The thickness of the silicone oil film was calculated by dividing the mass increase of the wafer (before and after spin coating) by the surface area of the wafer and the density of silicone oil. The slipperiness of the surface was evaluated by placing a 3 µL droplet of various liquids (water, coke, orange juice, milk or DMF) on the wafers and observing its sliding motion at a tilt angle of 10°. Optical micrographs were captured to visualise droplet movement and measure sliding velocity, which were used to confirm the effectiveness of the SLIPS.

**Tetracycline Encapsulation by Surface PISA**

Each POEGMA-grafted glass slip was immersed in a solution containing BzMA (70 mg, 0.4 mmol), Eosin Y disodium salt (0.07 mg, 0.0001 mmol) in ethanol (400 µL) followed by the addition of tetracycline (8 mg, 0.018 mmol). Surface PISA was conducted under LED light (λ_m_ = 405 nm; irradiance = 5 mW/cm^2^ at 5 cm distance) for 72 h at room temperature. After reaction, the surfaces were rinsed several times by ethanol and dried with nitrogen stream, followed by vacuum drying at room temperature.

**Antibacterial Assay of Tetracycline Encapsulated Surface Nanoparticles**

Fresh *S.aureus* suspensions were prepared by culturing the bacteria in LB broth until they reached a concentration of 8 × 10^6^ CFU/mL. Glass slips were sterilised by immersing them in ethanol and drying them with airflow. The bacterial suspension was uniformly spread onto agar plates using a cotton swab, ensuring even coverage. The inoculum was allowed to absorb into the agar for 5 min. The sterilised glass slips were then flipped and carefully placed onto the agar plates, ensuring good contact with the surface. The agar plates were incubated at 37 °C for 12 h. After incubation, the zones of inhibition (ZOI) were measured to evaluate the antibacterial activity. The original diameter of the glass slips was *D*_O_ (12 mm), and the longest diameter of ZOI was measured as *D*_L_, and the actual diameter (D*_L_-*D*_O_*) of the inhibition zone was defined as the “diameter of the zone of bacterial inhibition.”

**Statistical Analysis**

Statistical significance was determined by unpaired t-test: ***p ≤ 0.001, *p ≤ 0.05. Data are presented as mean ± s.d. n=3.


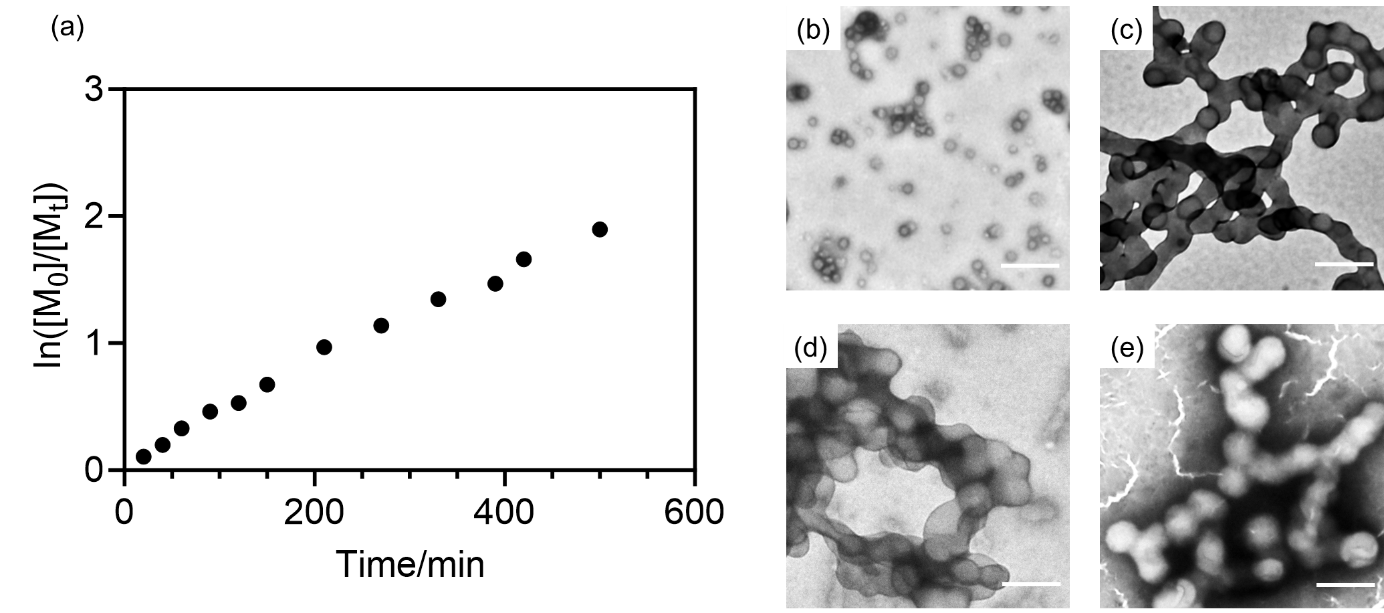


**Figure S1.** (a) Polymerisation kinetics of solution photo-PISA using POEGMA and BzMA. (b-e) TEM images of nanoparticles with various morphologies obtained by photo-PISA at polymerisation times of 20, 120, 210, and 500 minutes. Scale bar: 200 nm.


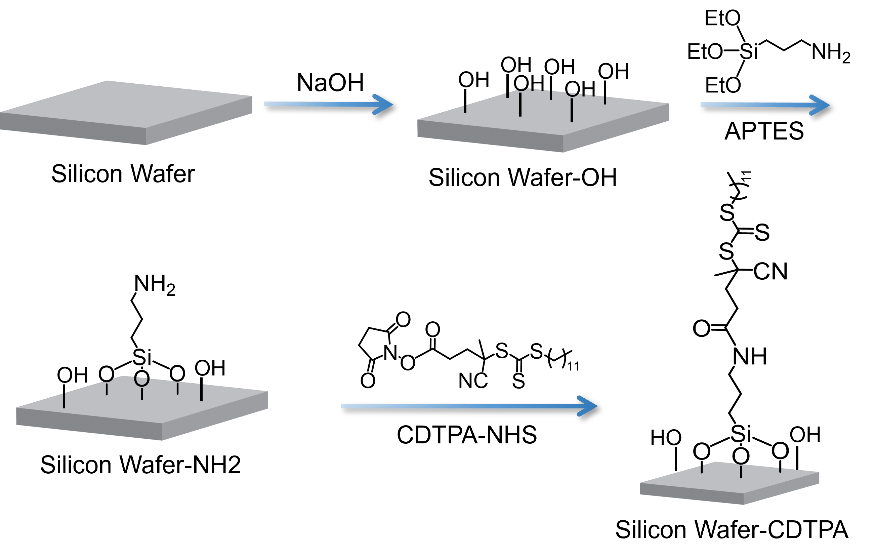


**Scheme S1.** Synthesis of surface-tethered RAFT agent CDTPA using silicon wafers as planar surface substrates.


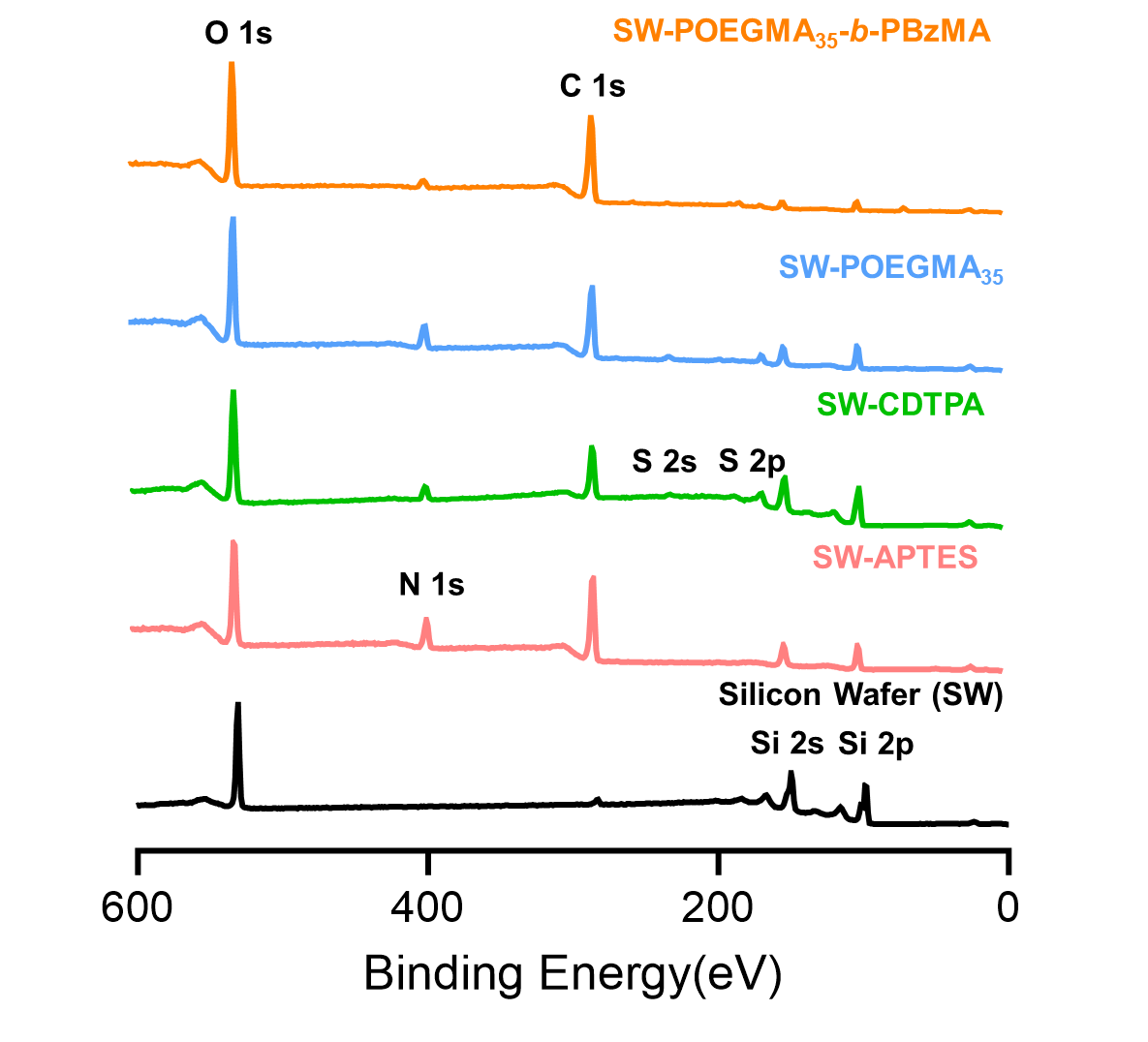


**Figure S2.** XPS survey spectra of bare and modified silicon wafers.


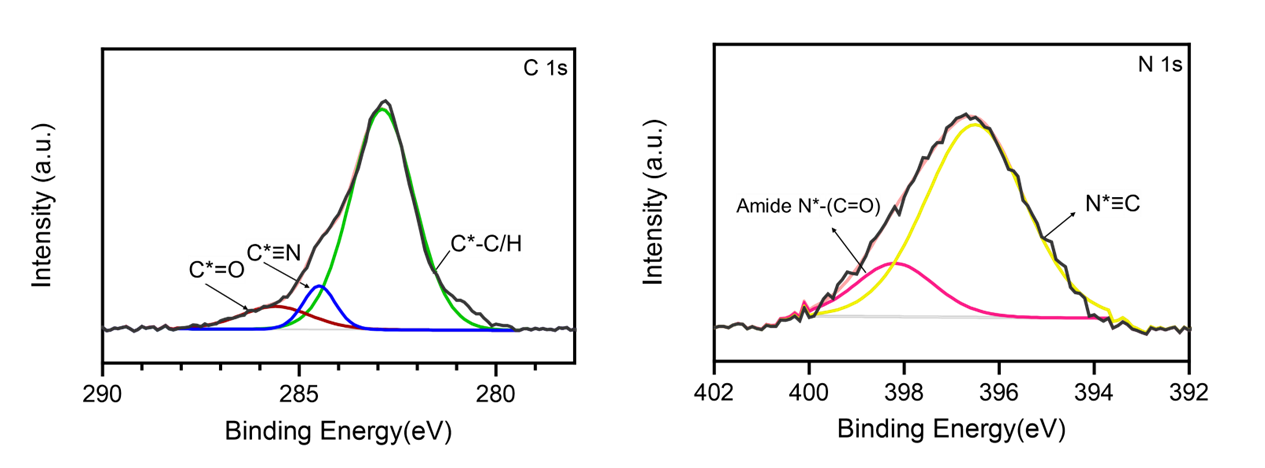
 **Figure S3.** Deconvolution of C 1s and N 1s XPS spectra of silicon wafers treated with 10 % APTES followed by CDTPA attachment.

**Table S1.** Molecular characteristics of the free POEGMA macro-CTA collected in the synthesis of surface-tethered POEGMA.

| Sample | Feed ratio | Conversion  (%)*^a^* | DP | *M*_n_*_,SEC_*  (g/mol)*^b^* | *M*_n,theo_  (g/mol)*^c^* | Dispersity  *(Đ)^b^* |
| --- | --- | --- | --- | --- | --- | --- |
| POEGMA-CDTPA35 | 50: 1 | 71 | 35 | 11930 | 10900 | 1.38 |
| POEGMA-CDTPA250 | 300: 1 | 84 | 250 | 69340 | 75400 | 1.56 |

Note: *^a^* determined by ^1^H NMR by comparing the integral of protons of the vinyl group and methylene adjacent to the ester group of the monomer before and after polymerisation; *^b^* determined by SEC using THF as eluent; *^c^* calculated by the equation *M*_n,theo_ = Conversion × *DP*_monomer_ × *M*_w_,_monomer_+ *M*_w,CTA_.


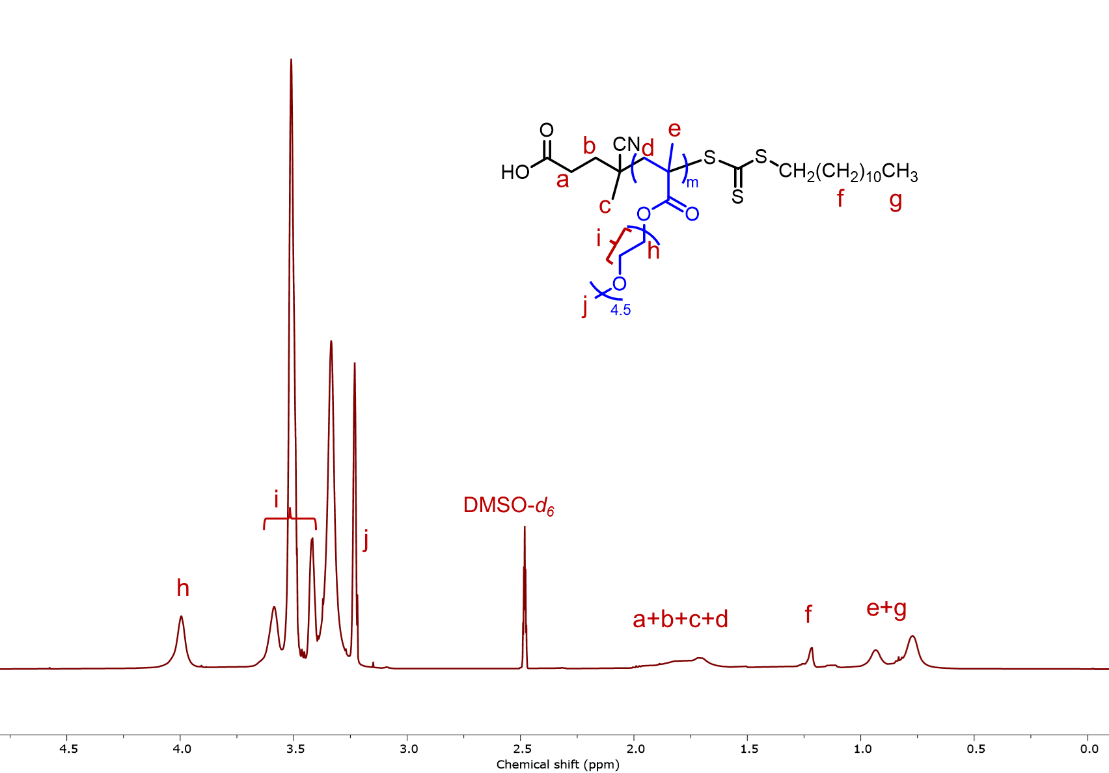


**Figure S4.** ^1^H NMR spectrum of pure free POEGMA-CDTPA in DMSO-d_6_.

**Table S2.** Apparent chemical surface composition in atomic percentage (At.%) of the silicon wafers.

| Sample | O 1s | C 1s | N 1s | S 2p | Si |
| --- | --- | --- | --- | --- | --- |
| Bare silicon wafer (SW) |  |  |  | - | - |
| SW-APTES10/TESPN90 (Low) | 23.90 | 38.81 | 7.58 | - | 29.71 |
| SW-APTES50/TESPN50 (Middle) | 24.76 | 34.03 | 8.68 | - | 32.53 |
| SW-APTES100/TESPN (High) | 22.74 | 52.63 | 9.43 | - | 15.20 |
| SW-APTES100-CDTPA (High) | 24.20 | 26.56 | 3.67 | 9.96 | 35.62 |
| SW-APTES100-CDTPA-POEGMA (High) | 26.39 | 47.98 | 8.61 | 2.88 | 14.14 |
| SW-APTES100-CDTPA-POEGMA-*b*-PBzMA (High) | 26.69 | 62.94 | 3.49 | 0.81 | 6.06 |


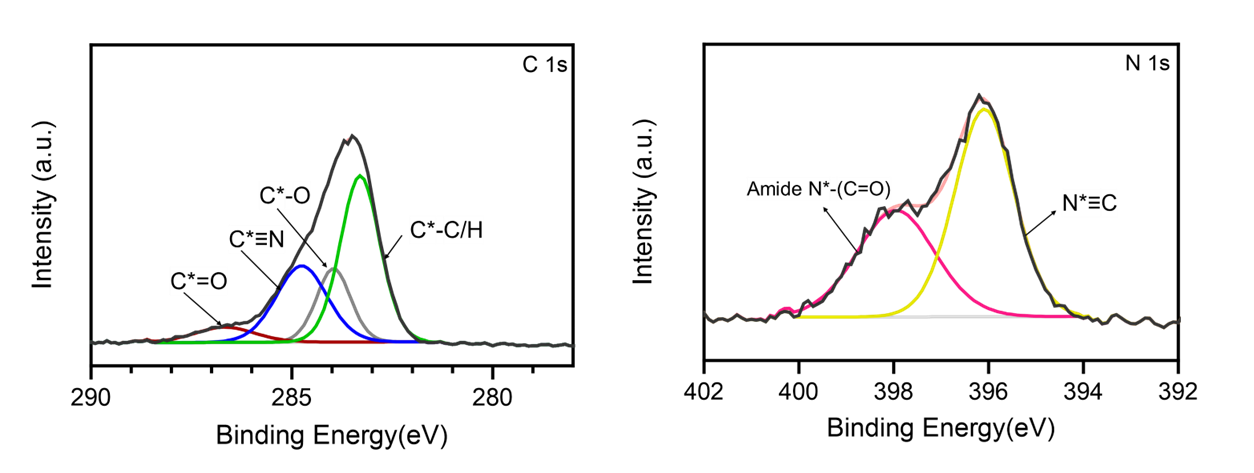


**Figure S5.** Deconvolution of C 1s XPS spectrum of surface-tethered POEGMA polymer brushes.


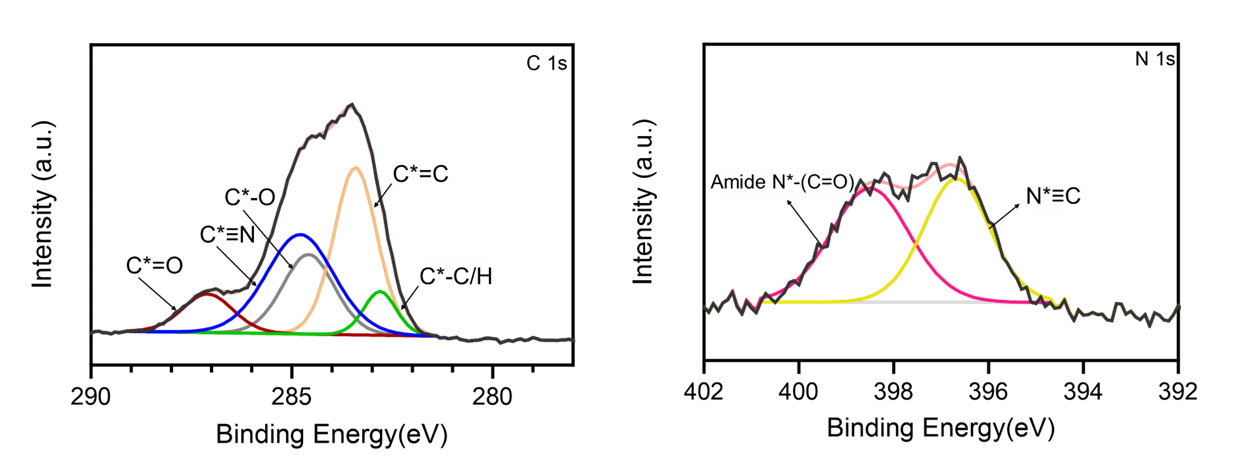


**Figure S6.** Deconvolution of C 1s XPS spectrum of POEGMA-*b*-PBzMA copolymer grafted onto a silicon wafer via surface PET-RAFT polymerisation.


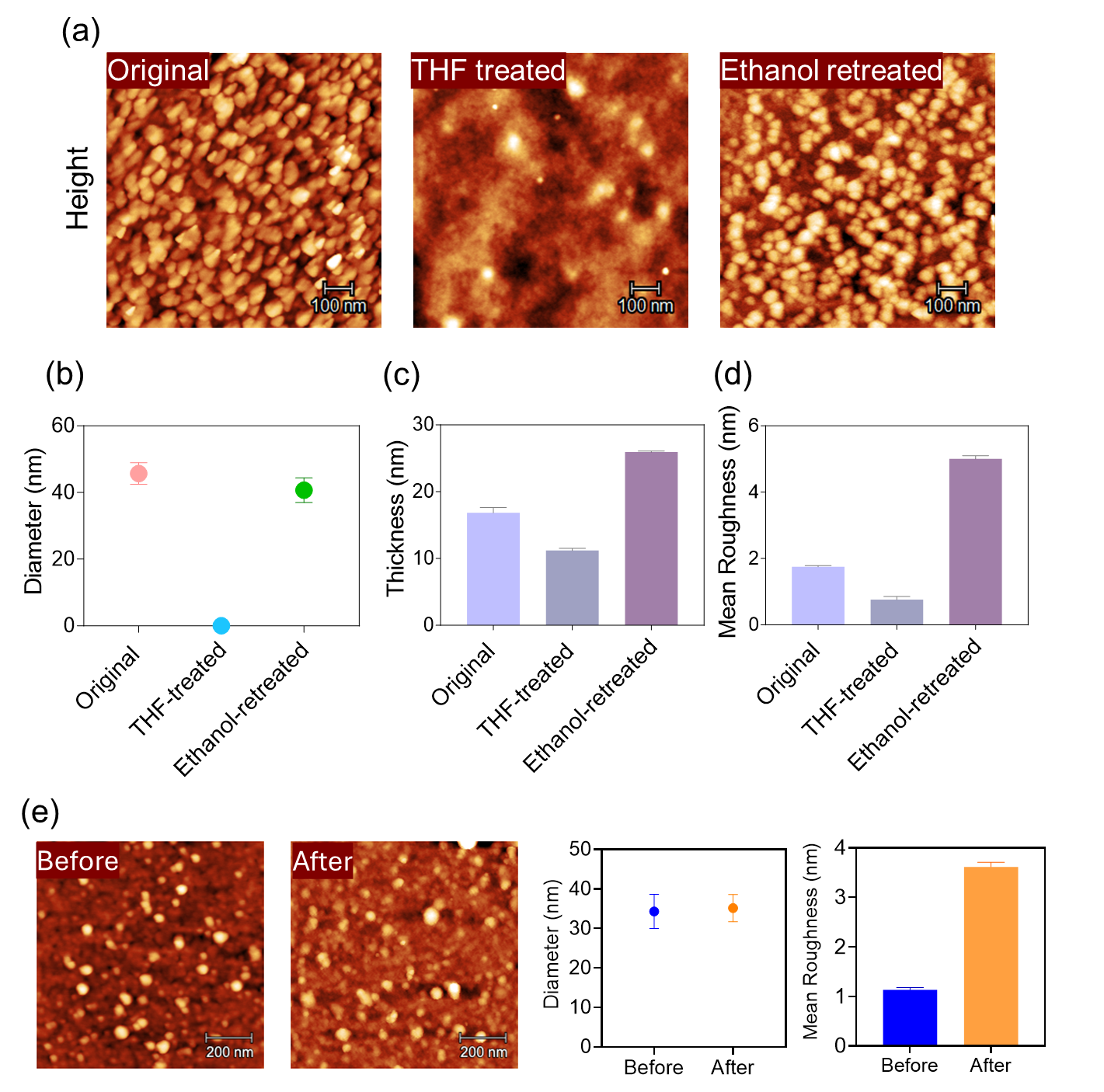


**Figure S7.** (a) AMF images, (b) diameter, (c) thickness and (d) mean roughness of the original surface PISA nanoparticles obtained after 48 h polymerization, surface PISA nanoparticles after treatment of THF and ethanol. (e) AFM images, particle size, and surface roughness of surface PISA nanoparticles before and after five cycles of alternating treatment of THF (10 min) and ethanol (30 min).


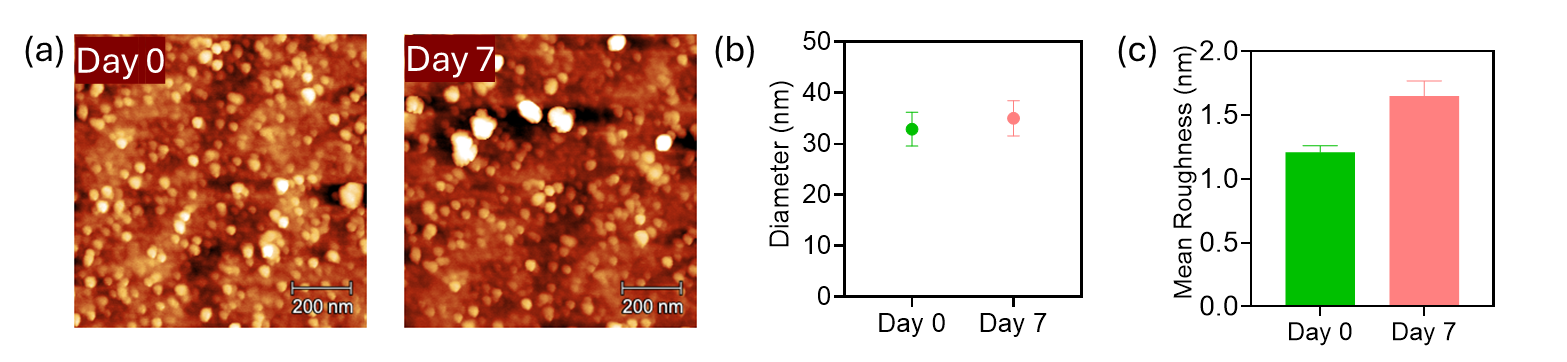


**Figure S8.** (a) AFM images, (b) particle size, and (c) surface roughness of surface PISA nanoparticles before and after alternating immersion in ethanol and water (one day each) over a 7-day period.


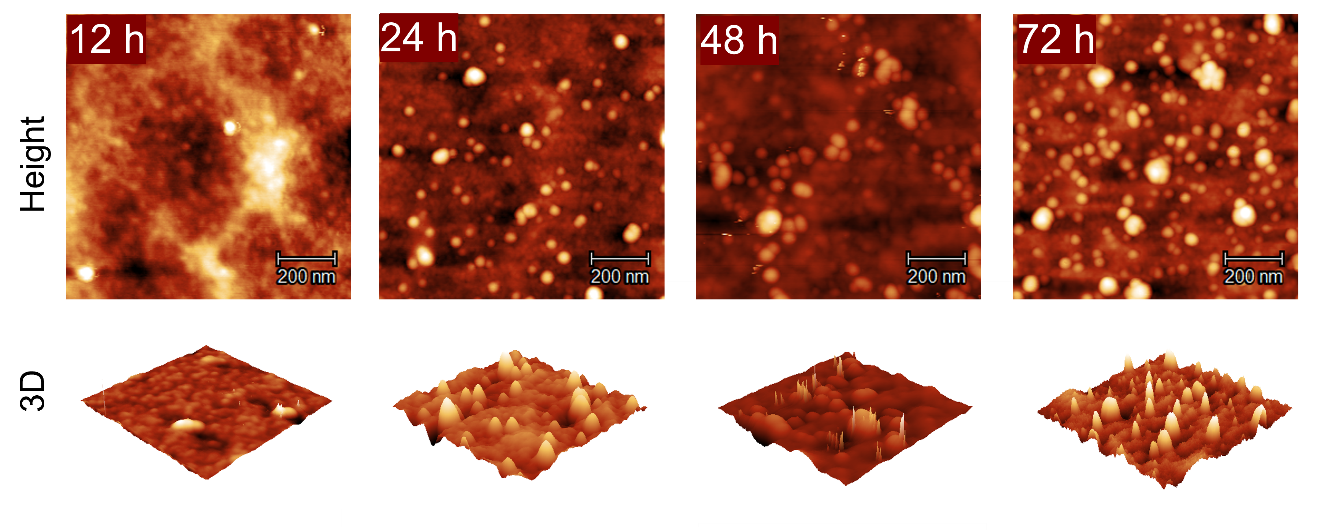


**Figure S9.** AFM images of surface PISA nanoparticles using DAAM as a core-forming monomer. Monomer concentration: 175 mg/mL. Solvent: water.


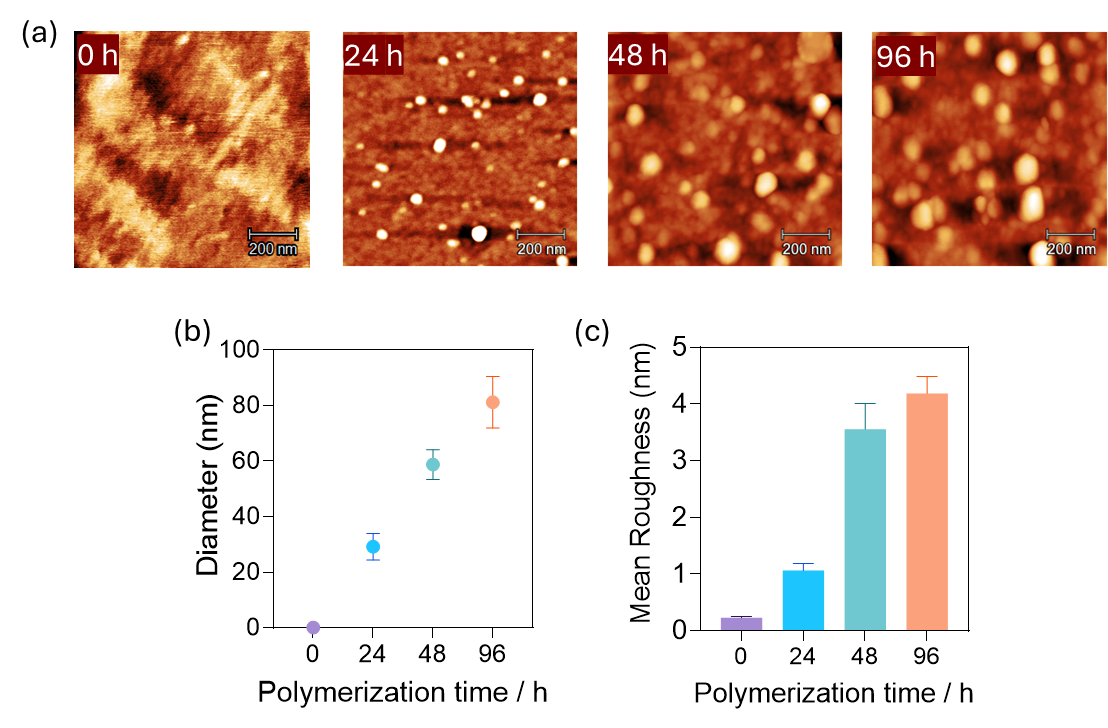


**Figure S10.** (a) AFM images, (b) particle size, and (c) surface roughness of surface PISA nanoparticles at different polymerization times using PDMA as a macro-RAFT agent and St as a core-forming monomer. Monomer concentration: 175 mg/mL. Solvent: ethanol.


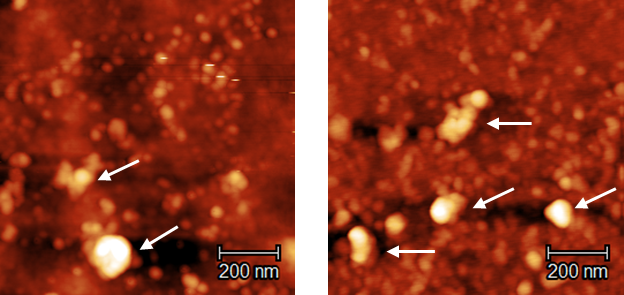


**Figure S11.** AFM images showing elongated and merged nanoparticles formed during surface PISA using BzMA.


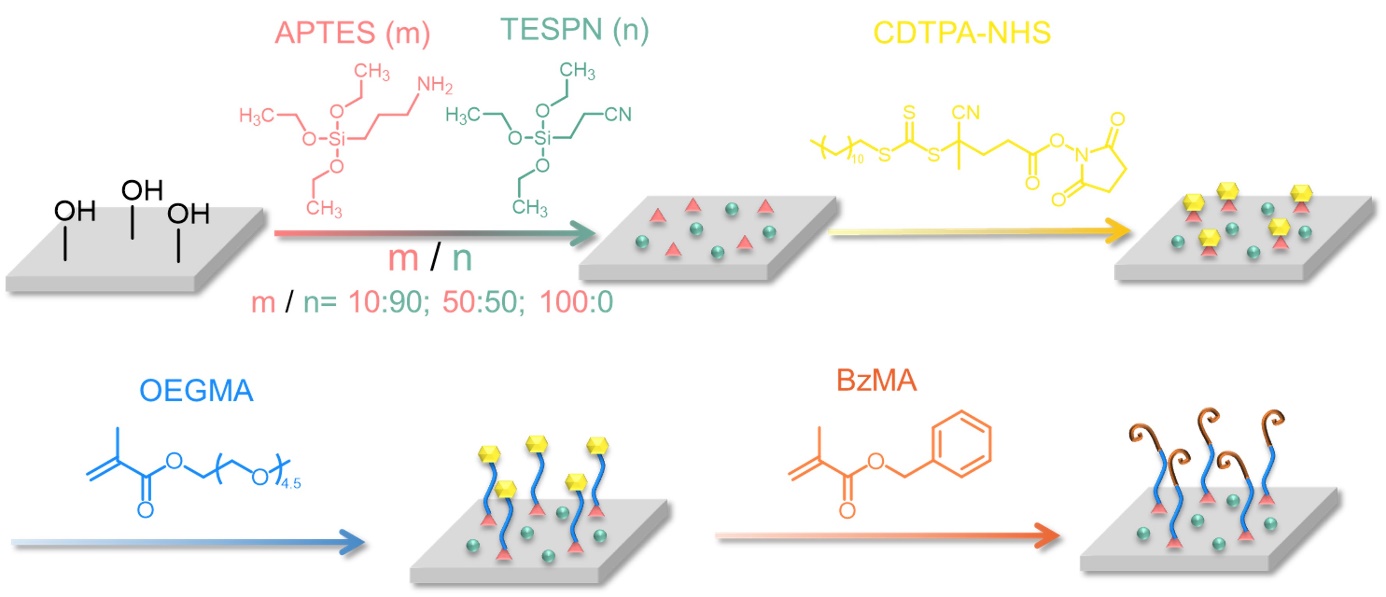


**Scheme S2.** Illustration of controlling the grafting density of surface-tethered POEGMA for surface PISA by varying the molar ratio of APTES and TESPN.


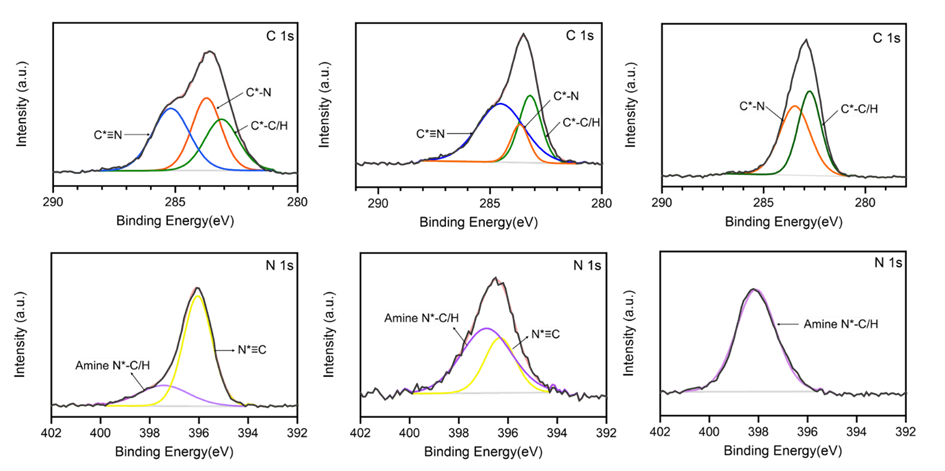


**Figure S12.** Deconvolution of C 1s and N 1s XPS spectra of silicon wafers treated with 10%:90% (left lane), 50%:50% (middle lane), and 100%:0% (right lane) molar ratios of APTES and TESPN.


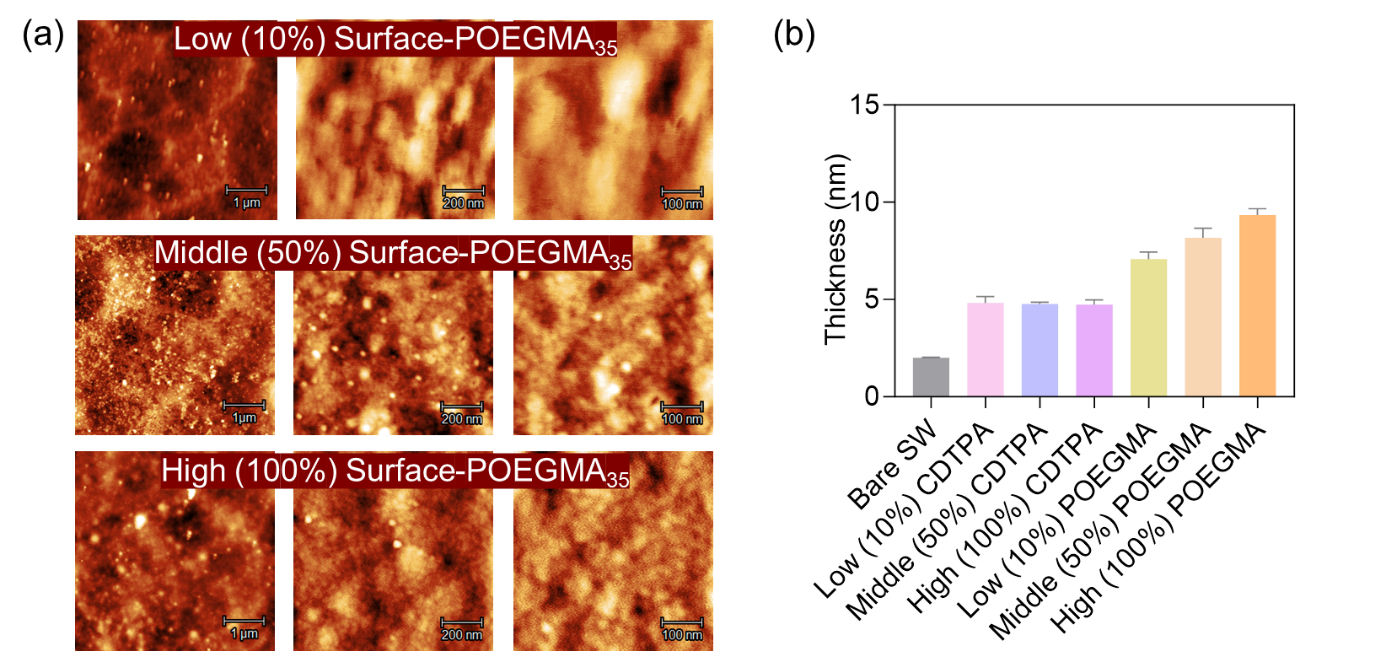


**Figure S13.** AFM images (a) and thickness (b) of surface-tethered POEGMA with low (10%), middle (50%), and high (100%) grafting density.


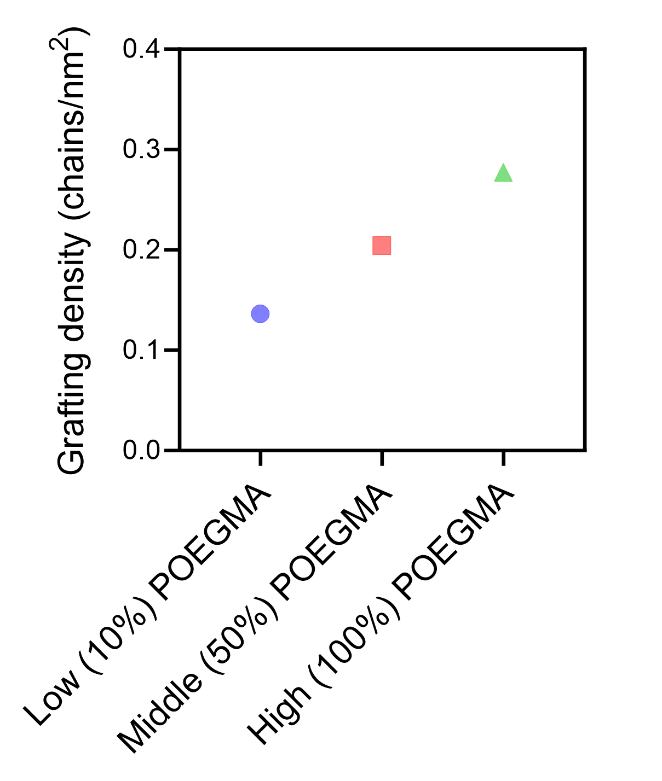


**Figure S14.** Grafting density of surface-tethered POEGMA brushes measured by ellipsometry.


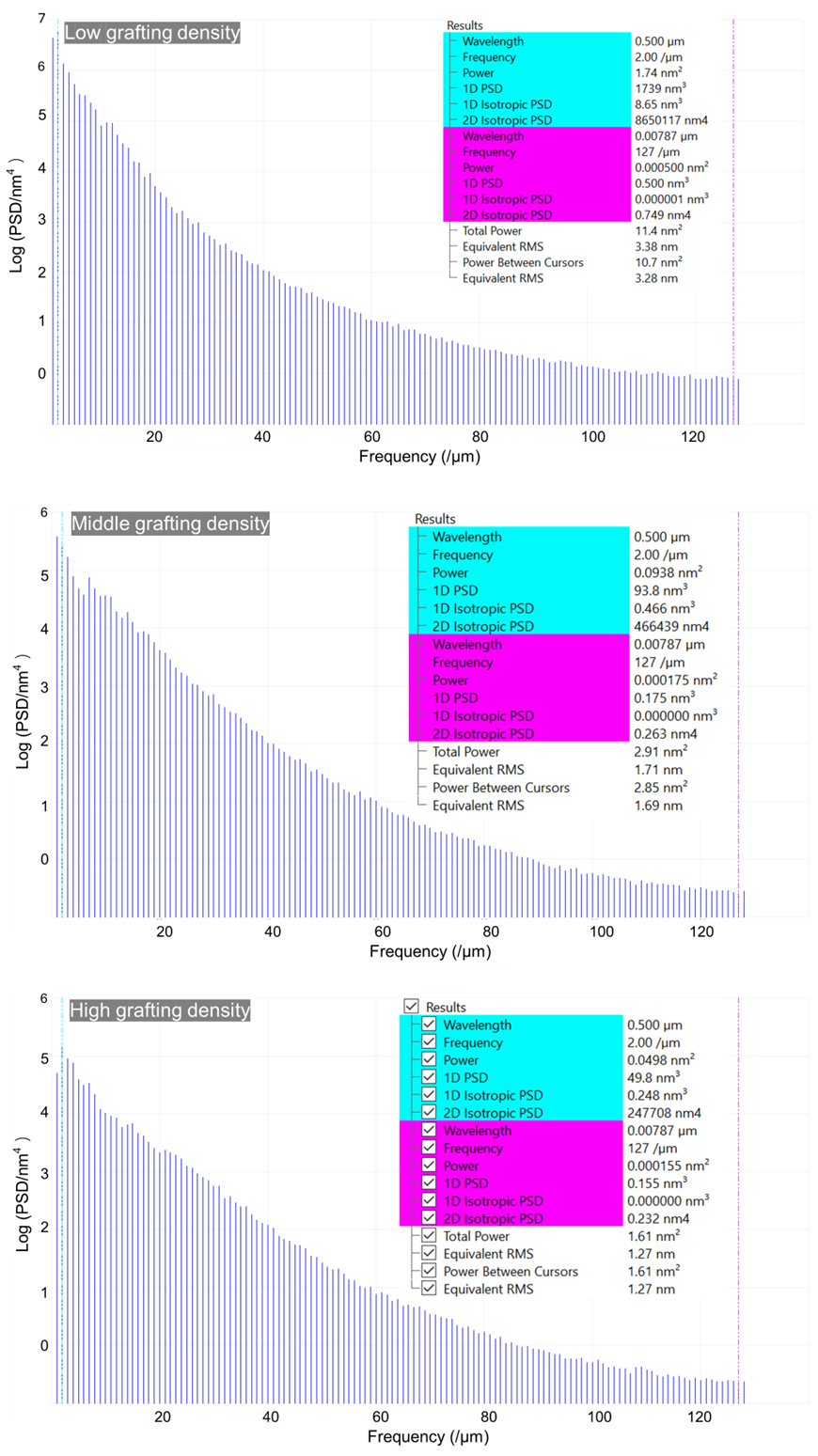


**Figure S15.** Power spectral density analysis of surface roughness based on the AFM images of surface PISA using surface-tethered POEGMA with low, middle, and high grafting density.


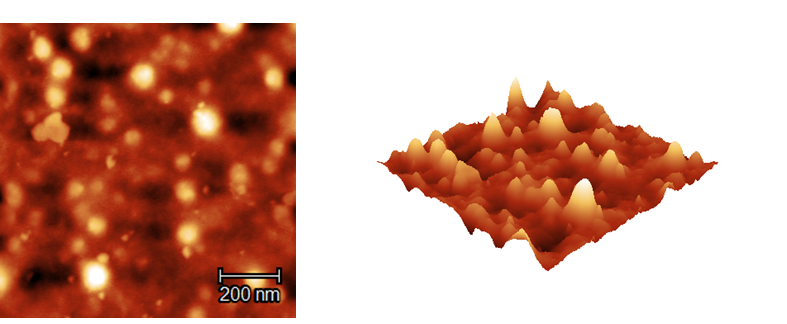


**Figure S16.** AFM images of the surface PISA mediated by surface-tethered POEGMA_250_ after reaction for 144 h.


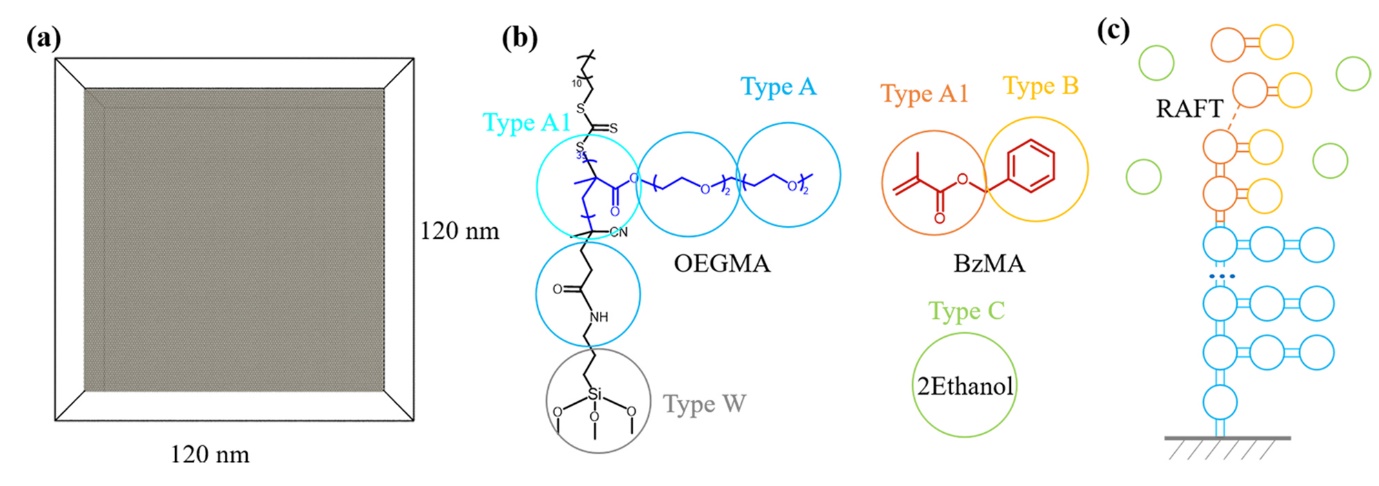


**Figure S17.** (a) The model of silicon wafer. (b) The models of OEGMA, BzMA and the solvent. (c) Illustration of DPD simulation method for surface PISA.


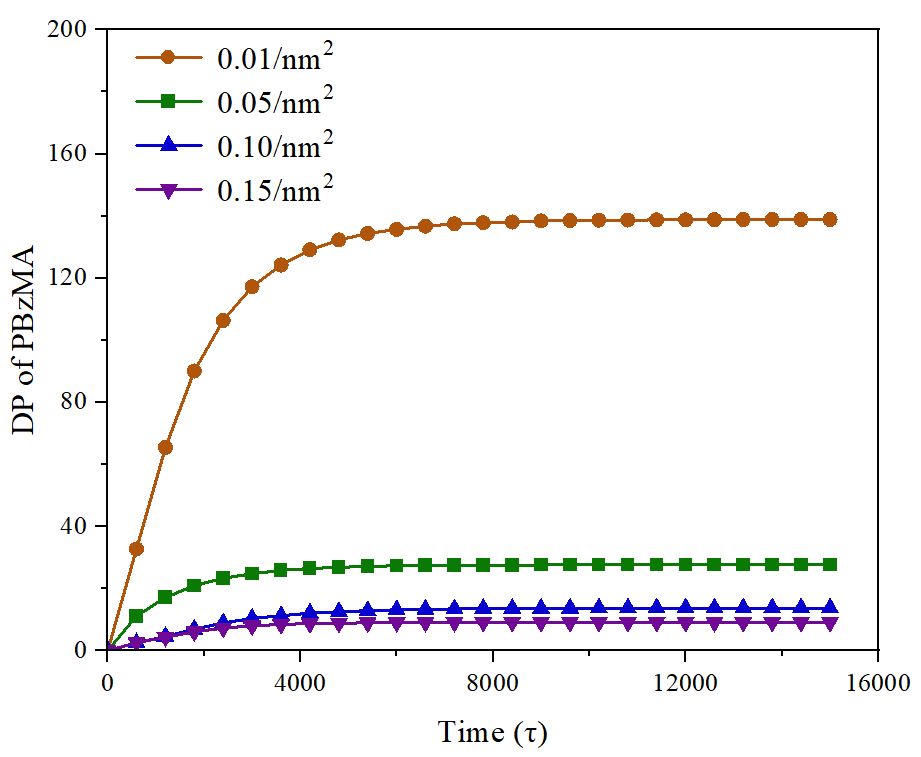


**Figure S18.** DP of PBzMA as a function of simulation time using different grafting densities of POEGMA.


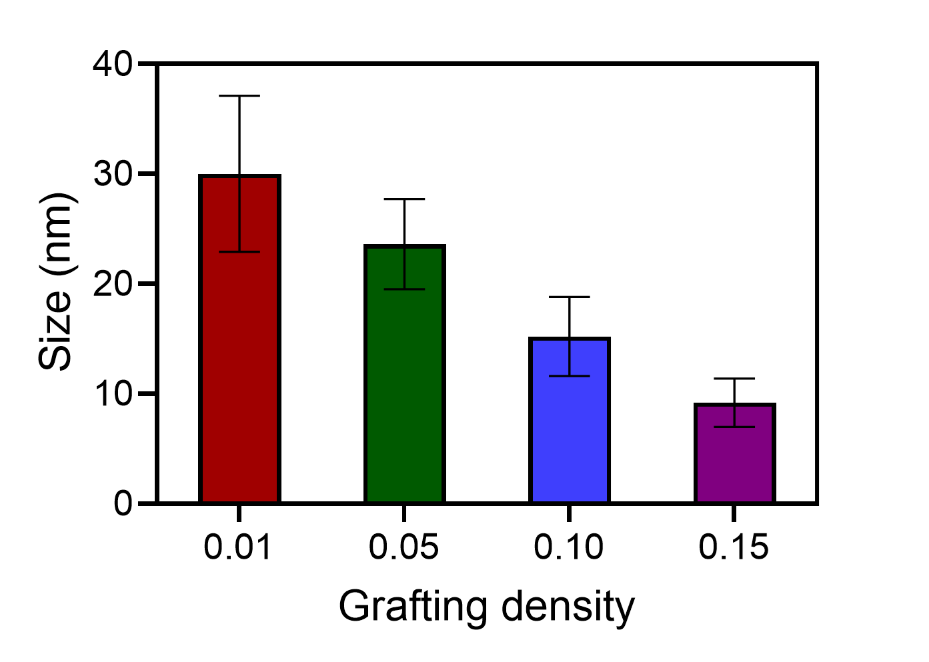


**Figure S19.** Average size of nanoparticles at the end of DPD simulation using different grafting densities of POEGMA.


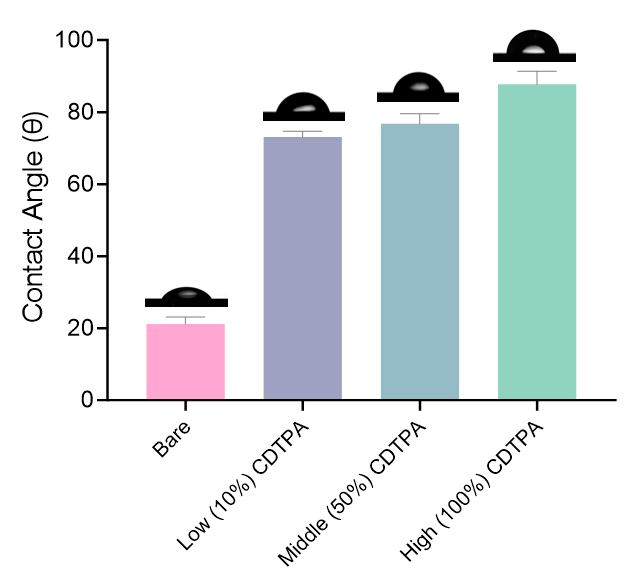


**Figure S20.** Contact angle measurement of silicon wafers modified with different amount of CDTPA.


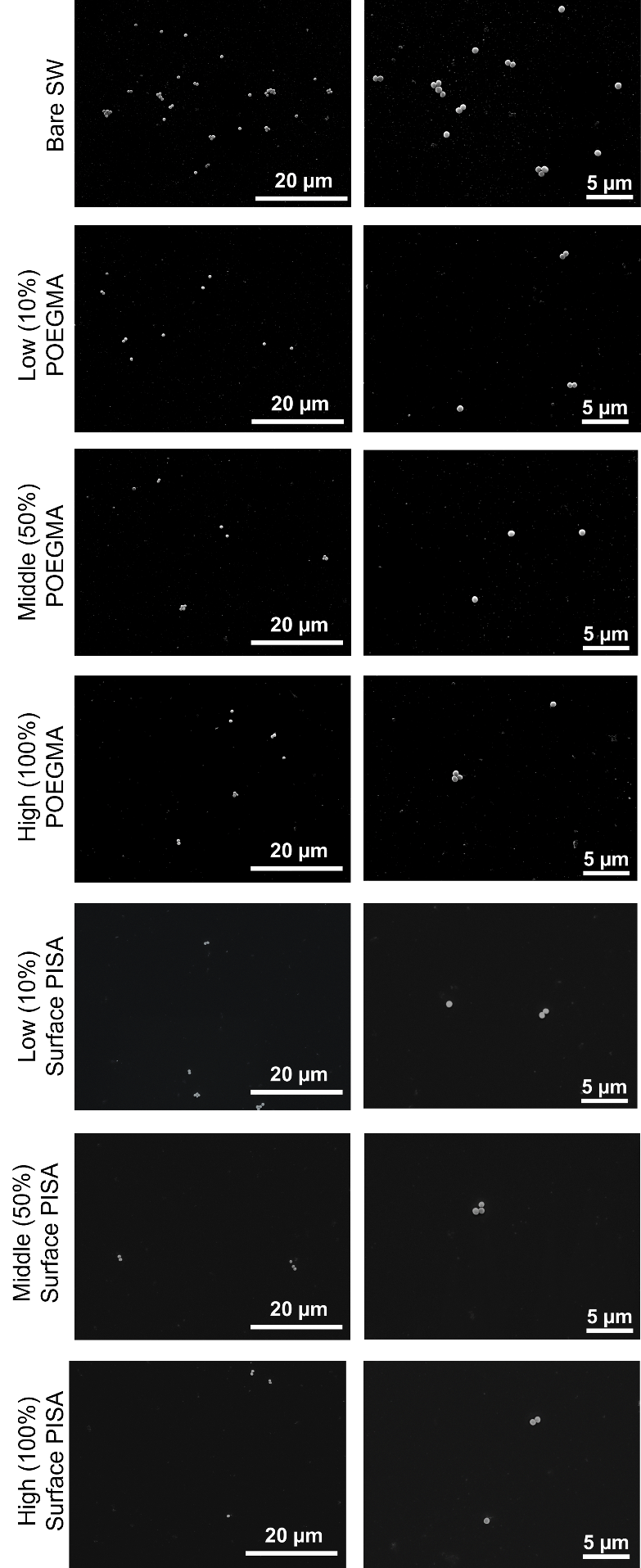


**Figure S21.** SEM images (scale bar: top panel 20 µm, bottom panel: 5 µm) of *S. aureus* adhered to the silicon wafer surfaces. The figure was re-organized from Figure 6b for improved clarity.


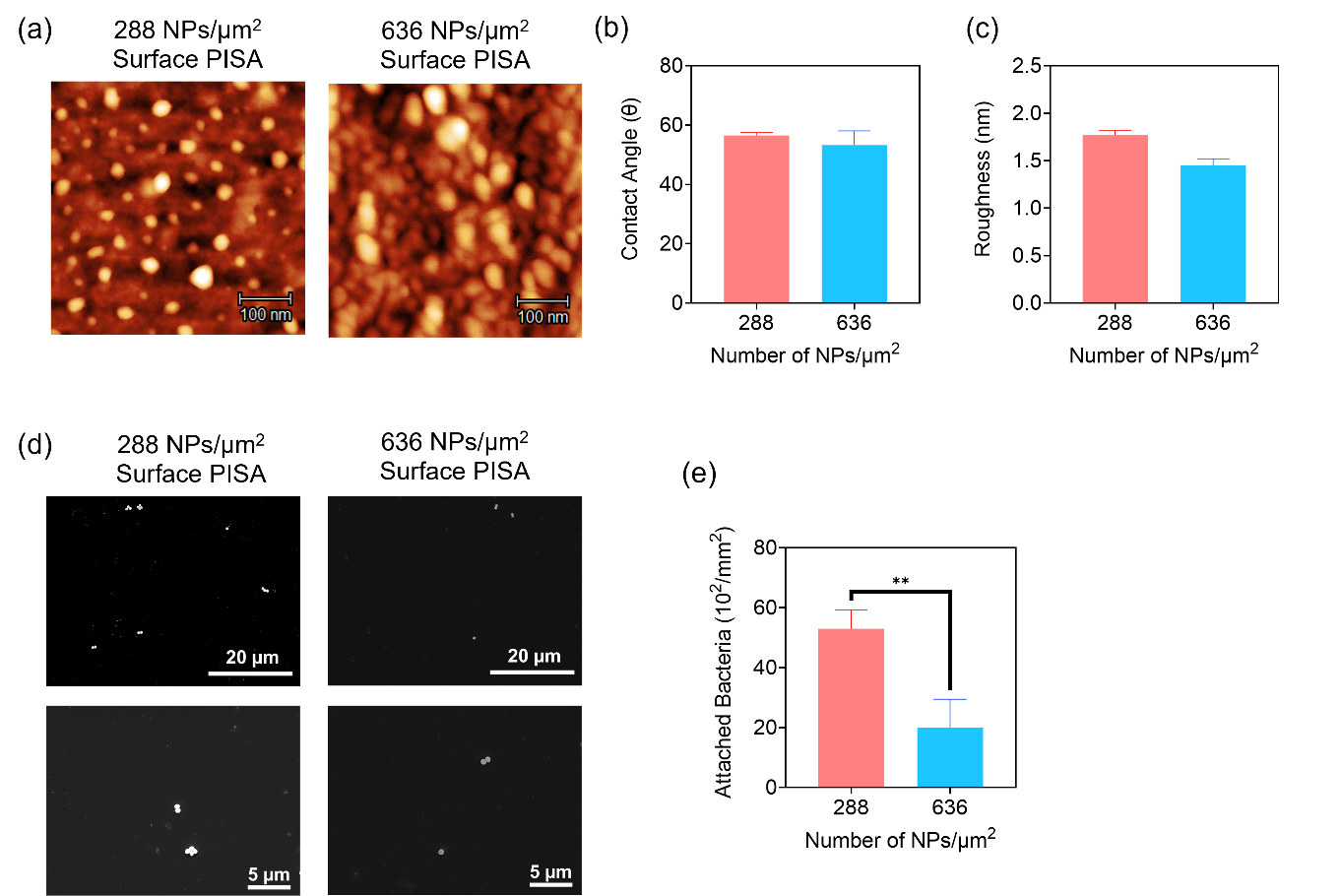


**Figure S22.** (a) AFM images of surfaces with low and high nanoparticle (NP) densities prepared via surface PISA. (b) Contact angles and (c) surface roughness of the two surfaces. (d) SEM images and (e) number of *S. aureus* adhered to the two surfaces.


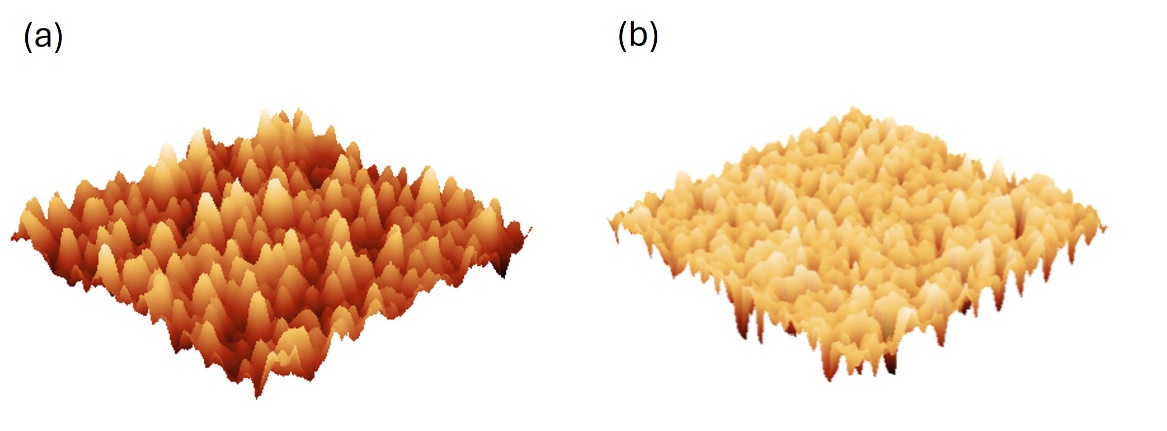


**Figure S23.** 3D AFM images (0.5 µm × 0.5 µm) of surface PISA (a) before and (b) after Au coating.


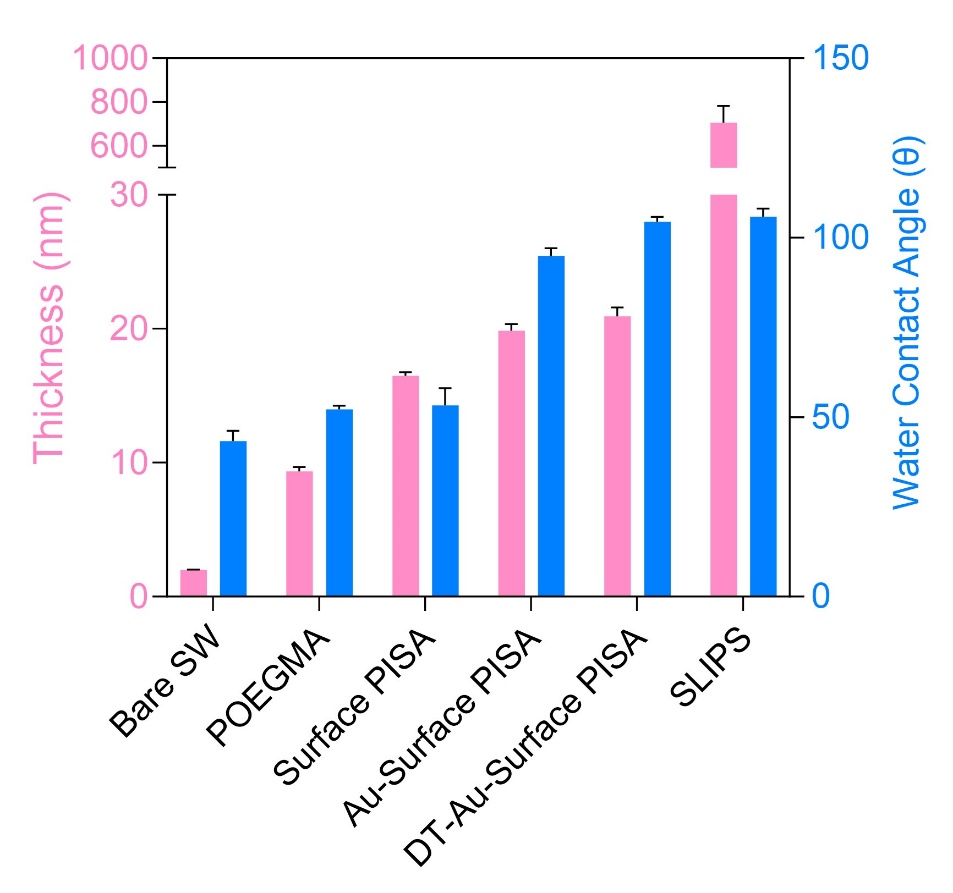


**Figure S24.** Thickness and water contract angle of different surfaces.


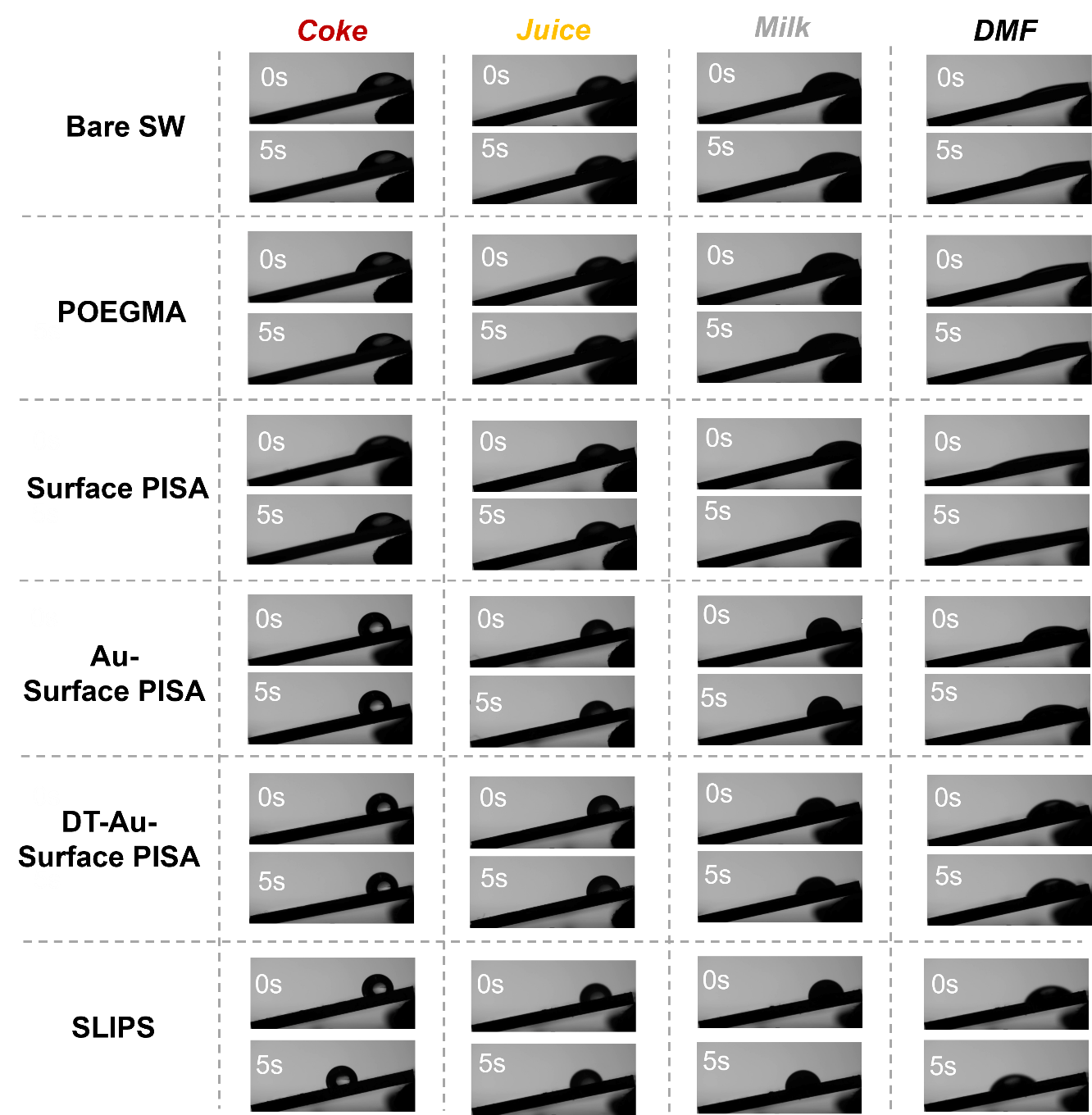


**Figure S25.** Images of droplets of various liquids (e.g., Coke, orange juice, milk, and DMF) sliding off different surfaces at a tilt angle of 10^o^.


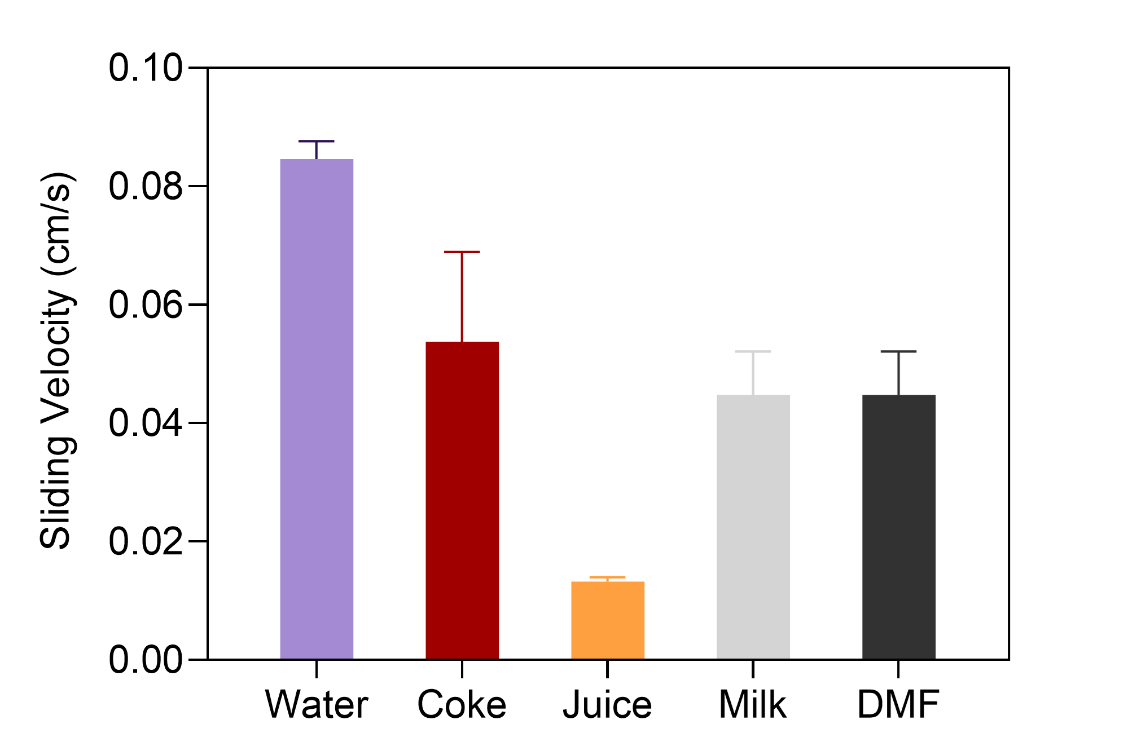


**Figure S26.** Sliding velocity of various liquid droplets on the SLIPS at a tilt angle of 10^o^.


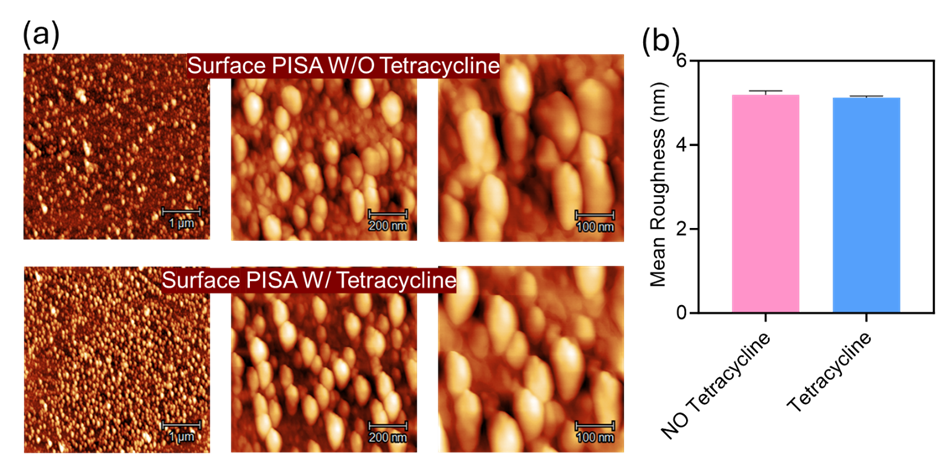


**Figure S27.** AFM images and mean surface roughness of the surface PISA with and without the addition of tetracycline.

**References**

[1] X. Xu, X. Huang, Y. Chang, Y. Yu, J. Zhao, N. Isahak, J. Teng, R. Qiao, H. Peng, C.-X. Zhao, T. P. Davis, C. Fu, A. K. Whittaker, *Biomacromolecules* **2021**, *22*, 330-339.

[2] R. D. Groot, P. B. Warren, *J. Chem. Phys.* **1997**, *107*, 4423-4435.

[3] J. D. Weeks, D. Chandler, H. C. Andersen, *J. Chem. Phys.* **1971**, *54*, 5237-5247.

[4] C. M. Hansen, *Danish Technical: Copenhagen* **1967**, *14*.

[5] H. Liu, Y.-L. Zhu, Z.-Y. Lu, F. Müller-Plathe, *J. Comput. Chem.* **2016**, *37*, 2634-2646.

[6] Y.-L. Zhu, C.-L. Fu, Z.-W. Li, Z.-Y. Sun, *J. Phys. Chem. Lett.* **2020**, *11*, 179-183; X.-K. Yu, H.-Y. Zhao, J.-P. Li, X.-J. Li, J.-Q. Yang, Y.-L. Zhu, Z. Lu, *J. Phys. Chem. Lett.* **2022**, *13*, 7087-7093.

[7] Y.-L. Zhu, H. Liu, Z.-W. Li, H.-J. Qian, G. Milano, Z.-Y. Lu, *J. Comput. Chem.* **2013**, *34*, 2197-2211; Y.-L. Zhu, D. Pan, Z.-W. Li, H. Liu, H.-J. Qian, Y. Zhao, Z.-Y. Lu, Z.-Y. Sun, *Mol. Phys.* **2018**, *116*, 1065-1077.

[8] O. O. Oyeneye, W. Z. Xu, P. A. Charpentier, *RSC advances* **2015**, *5*, 76919-76926; D. S. Benoit, S. Srinivasan, A. D. Shubin, P. S. Stayton, *Biomacromolecules* **2011**, *12*, 2708-2714.
